# Charge migration and charge transfer in molecular systems

**DOI:** 10.1063/1.4996505

**Published:** 2017-12-27

**Authors:** Hans Jakob Wörner, Christopher A. Arrell, Natalie Banerji, Andrea Cannizzo, Majed Chergui, Akshaya K. Das, Peter Hamm, Ursula Keller, Peter M. Kraus, Elisa Liberatore, Pablo Lopez-Tarifa, Matteo Lucchini, Markus Meuwly, Chris Milne, Jacques-E. Moser, Ursula Rothlisberger, Grigory Smolentsev, Joël Teuscher, Jeroen A. van Bokhoven, Oliver Wenger

**Affiliations:** 1 Laboratory of Physical Chemistry, ETH Zürich, Zürich, Switzerland; 2 Laboratory of Ultrafast Spectroscopy and Lausanne Centre for Ultrafast Science (LACUS), École Polytechnique Fédérale de Lausanne, Lausanne, Switzerland; 3 Department of Chemistry, University of Fribourg, Fribourg, Switzerland; 4 Institute of Applied Physics, University of Bern, Bern, Switzerland; 5 Department of Chemistry, University of Basel, Basel, Switzerland; 6 Department of Chemistry, University of Zürich, Zürich, Switzerland; 7 Department of Physics, ETH Zürich, Zürich, Switzerland; 8 Department of Chemistry, University of California, Berkeley, California 94720, USA; 9 Institute of Chemical Sciences and Engineering, École Polytechnique Fédérale de Lausanne, Lausanne, Switzerland; 10 Department of Physics, Politecnico di Milano, 20133 Milano, Italy; 11 SwissFEL, Paul-Scherrer Institute, Villigen, Switzerland; 12 Paul-Scherrer Institute, Villigen, Switzerland; 13 Institute for Chemical and Bioengineering, ETH Zürich, Zürich, Switzerland

## Abstract

The transfer of charge at the molecular level plays a fundamental role in many areas of chemistry, physics, biology and materials science. Today, more than 60 years after the seminal work of R. A. Marcus, charge transfer is still a very active field of research. An important recent impetus comes from the ability to resolve ever faster temporal events, down to the attosecond time scale. Such a high temporal resolution now offers the possibility to unravel the most elementary quantum dynamics of both electrons and nuclei that participate in the complex process of charge transfer. This review covers recent research that addresses the following questions. Can we reconstruct the migration of charge across a molecule on the atomic length and electronic time scales? Can we use strong laser fields to control charge migration? Can we temporally resolve and understand intramolecular charge transfer in dissociative ionization of small molecules, in transition-metal complexes and in conjugated polymers? Can we tailor molecular systems towards specific charge-transfer processes? What are the time scales of the elementary steps of charge transfer in liquids and nanoparticles? Important new insights into each of these topics, obtained from state-of-the-art ultrafast spectroscopy and/or theoretical methods, are summarized in this review.

## I. INTRODUCTION

The spatial redistribution of electronic charge in molecules and condensed matter belongs to the most important primary events in all photoinduced processes in chemical and biological systems and in materials (see Ref. 1 and references therein). Oxidation and reduction reactions, photoinduced charge transfer (CT) in metal complexes and charge injection in solar cells are a few examples of such charge transfer processes. Understanding these dynamics at the atomic level generally requires a very high temporal resolution in the femtosecond range when individual vibrational motions must be resolved and even in the attosecond domain to visualize electronic motion. This level of understanding is essential, not only to achieve fundamental insights into these processes, but also on a longer perspective for improving applications relying on charge transfer. The ability to measure the time scales of the primary processes responsible for charge transfer indeed offers the potential for optimizing the efficiency of a charge-transfer system by accelerating the desired pathway and slowing down the competing ones. Such work can be expected to lead to more selective catalysts, better energy- and/or data-storage systems and more efficient photovoltaic systems.

Owing to its importance from both fundamental and applied perspectives, the field of charge transfer has attracted continued interest from scientists over more than 80 years. The first comprehensive theory of charge transfer has been formulated by R. A. Marcus in Refs. 2 and 3, which has led to the award of the Nobel prize in 1992. Marcus theory is a classical rate-law theory that is conceptually related to Eyring's transition state theory. Marcus theory has been remarkably successful in describing many different types of charge transfer, i.e., intramolecular, intermolecular, metal-to-ligand, ligand-to-metal, and charge transfer to solvent (CTTS). At the time when Marcus formulated his theory, time-resolved measurements had just reached the nanosecond level, owing to the work of Eigen, Norrish, and Porter, who were awarded the Nobel prize in 1967. Over the following 50 years, the time resolution has continuously increased. Reaching the femtosecond time scale made the resolution of individual vibrations possible.^4^ Shortly after the Nobel Prize was awarded to Zewail in 1999 for the development of femtochemistry, the first experiments with attosecond laser pulses were reported,^5,6^ marking the beginning of attosecond science. The shortest pulse duration reported to date amounts to only 43 attoseconds.^276^ Whereas femtosecond time-resolved experiments are widely applied to study charge-transfer problems, as also shown in this review, this area has only recently moved into the focus of attosecond spectroscopy. One experiment has measured a ∼4 fs quantum beat in a photofragmentation signal associated with electronic dynamics in the phenylalanine cation,^7^ and the other has described the reconstruction and control of attosecond (i.e., sub-femtosecond) charge migration in the iodoacetylene cation.^8^

Here, we review some recent advances in understanding charge-transfer processes ranging from attosecond to microsecond time scales. We start by reviewing the basic concepts underlying charge transfer. The most fundamental process is purely electronic charge migration, i.e., a time-dependent oscillation of the charge density in a molecule, which is driven by the electronic coherence of a superposition state. We follow the convention introduced in previous work^9^ and designate the purely electronic dynamics as *charge migration*, whereas the dynamics involving nuclear motion is designated as *charge transfer*. In our usage, the term “charge migration” includes all types of purely electronic dynamics arising from electronic coherence, in addition to those where electron correlations beyond mean-field theories play a role.^9^

Charge migration on the attosecond time scale has recently been observed experimentally in the iodoacetylene cation using high-harmonic spectroscopy.^8^ The dynamics of an electron hole have been reconstructed with atomic spatial and attosecond temporal resolution. Strong-field laser control over attosecond charge migration has also been demonstrated. In particular, it has been shown that the location and the shape of the electron hole created by strong-field ionization can be controlled through the spatial orientation of the molecule with respect to the ionizing laser field. Moreover, non-adiabatic laser-driven electronic dynamics on a temporal scale of ∼1 fs have been demonstrated, establishing the possibility to extensively control charge migration on its natural time scale.

The dynamics of charge migration in the iodoacetylene cation are also discussed from a theoretical point of view by using Ehrenfest molecular-dynamics (MD) based on time-dependent density-functional theory (TDDFT). In this work, the dynamics of HCCI^+^ initially prepared in a superposition of its two lowest electronic states has been calculated including all electronic and vibrational degrees of freedom. The evaluation of the time-dependent electric dipole moment of the system is indeed entirely dominated by the expected frequency corresponding to the energetic separation of the two populated electronic states. These experimental and theoretical results on charge migration firmly establish the existence of a purely electronic and therefore sub-femtosecond mechanism for the spatial rearrangement of electronic charges in a molecule. The practical implications of this new mechanism for charge transfer on slower time scales have not yet been fully explored.

We then turn from charge migration to intramolecular charge transfer (ICT) and discuss the relaxation dynamics of the ethylene cation following ionization by an extreme-ultraviolet (XUV) attosecond pulse train (APT).^10^ A time-resolved experiment, realized in combination with a femtosecond near-infrared (IR) laser pulse, provided an improved isomerization time for the reaction $H_{2}C={\text{CH}}_{2}^{+}\to \text{HC}-{\text{CH}}_{3}^{+}$ of 30 ± 3 fs, and by comparison with *ab-initio* calculations the relevant conical intersections (CIs) could be identified.

We then discuss ligand-to-metal charge-transfer (LMCT) processes in metal complexes. This process has been observed in solvated ferric [Fe(CN)_6_]^3−^ complexes by means of time-resolved photoelectron spectroscopy of solutions^11,12^ and transient infrared absorption spectroscopy.^12^ The reduced iron center has been found to occur within the temporal resolution of the experiment (∼80 fs), whereas the back electron transfer was observed to take ∼500 fs in water and ∼1.2 ps in ethylene glycol.^12^ Intramolecular charge-transfer processes in polar solvents can only be accurately described when the participation of the solvent molecules is taken into account explicitly. The response of the solvent has been found to systematically assist the formation of the charge-separated states by energetically stabilizing the localized charges. Several molecular complexes exhibiting charge-transfer states have been studied and have led to the conclusion that the solvent molecules cannot be considered to be mere spectators of the charge transfer, but instead significantly modulate the process.

The reverse process is metal-to-ligand charge transfer (MLCT). Transient and static X-ray absorption spectroscopy (XAS) of *fac*-[ReBr(CO)_3_(bpy)] and related compounds revealed the existence of a two-center charge transfer process in the excitation of the low-lying electronically excited states, involving both the Re center and the Br ligand.^13^ The appearance of a pre-edge absorption feature at both the Re L_3_- and Br K-edges, which is absent from the ground state spectrum, is associated with the creation of a hole in the highest occupied molecular orbital following photoexcitation. Since both features display the same dynamics, the low-lying excited states are associated with a two-center charge transfer process from both the Re and Br centers. The role of solvent dynamics in MLCT excitation and relaxation processes was uncovered with atomistic resolution in classical MD calculations.^14^ The excitation of [Fe(bpy)_3_]^2+^ in an explicit water solvent from its low-spin state to its ^1,3^MLCT state, followed by the electronic relaxation to the high-spin (HS) state was simulated. Using different validated force fields for each of these three states, the structural dynamics of the solvation shell were investigated. This study showed an electronically driven decrease of the degree of solvation upon MLCT excitation. The relaxation from the MLCT to the HS state was found to occur on the sub-picosecond time scale, while thermalization occurs on the ps time scale, in agreement with recent experimental results.

We then turn from intramolecular to intermolecular charge transfer and discuss the first observation of a long-lived charge-separated state with two electrons accumulated on the charge acceptor in a molecular system, a pentad comprising a central anthraquinone (AQ) acceptor flanked by two $\text{Ru}{\left(\text{bpy}\right)}_{3}^{2+}$ photosensitizers and two peripheral triarylamine (TAA) donors.^15,16^ The availability of two redox equivalents and the long lifetime of the charge-separated state (870 ns) make this system attractive for artificial photosynthesis, such as CO_2_ reduction or water oxidation. Importantly, this system works without the use of sacrificial reagents. Detailed studies of the dynamics of this system by transient IR and ultraviolet (UV)-Vis spectroscopy in solution revealed that in ∼15% of all excited molecules, AQ is converted to its hydroquinone form (AQH_2_) via reversible intra-molecular electron transfer from the two TAA units (*τ* = 65 ps), followed by inter-molecular proton transfer from p-toluene sulfonic acid on a nanosecond time scale. A sequence of electron- and proton-transfer steps results in the AQH_2_ photoproduct that subsequently decays via proton-coupled electron transfer (PCET) with a time constant of 4.7 *μ*s.

Charge transfer within proteins plays a particularly important role in biological mechanisms. Here, we discuss charge transfer between amino-acid residues and porphyrin units in myoglobin (Mb), revealed by two-dimensional deep-UV spectroscopy.^17,18^ Specifically, it was shown that partial electron transfer in Mb takes place from Trp14 to the porphyrin unit, while relaxation of the more distant Trp7 unit is dominated by Förster resonant energy transfer (FRET). These results also show that the decay mechanism by electron transfer is general to all myoglobins.

Charge transfer in conjugated materials is particularly interesting in view of photovoltaic applications. Poly[N-9-hepta-decanyl-2,7-carbazole-alt-5,5‐(4,7-di-2-thienyl-2,1,3-benzothiadiazole)] (PCDTBT) belongs to the class of alternating donor-acceptor copolymers with high photovoltaic efficiency. The charge-transfer relaxation of PCDTBT and its constituent units have been studied using femtosecond transient absorption (TA) spectroscopy in solution and in the solid state. The charge-transfer character has been found to increase progressively from the initial moderately polar excited state, through torsional motion and solvent reorganization.^19^ Similar dynamics in solid CDTBT may be responsible for charge separation. Another class of systems that plays a crucial role in photovoltaics, in addition to donor-acceptor systems such as PCDTBT, is donor-bridge-acceptor systems. These systems, in particular, those containing a conjugated bridge are presently the most efficient systems in dye-sensitized solar cells (DSSCs) owing to their high extinction coefficients and wide spectral tunability through chemical substitution. Femtosecond time-resolved studies have been performed to measure charge-transfer dynamics within these molecules and into transition-metal-oxide substrates.^20^ These studies were backed by density-functional-theory (DFT) calculations. The dependence of the charge-carrier dynamics on the donor-acceptor distance, the conjugation length and the coupling of the dye with TiO_2_ was systematically studied by varying the chemical composition of the dye. A direct relation between the charge-transfer dynamics and the photovoltaic performance of DSSCs was found, highlighting the importance of molecular design in photovoltaics.

Charge transfer in photocatalytic systems is another very important process that plays a role, e.g., in solar hydrogen production from sunlight. Using X-ray absorption spectroscopy, evidence for the existence of a Co^*I*^ intermediate of cobaloxime has been obtained, and its solution structure determined.^21,22^ The distances from the cobalt atom to the nearest ligands were determined, as well as the displacement of the cobalt atom out of the plane formed by the planar ligands.

Charge transfer to solvent (CTTS) is another very important fundamental process that plays a role in biology, solution chemistry and electrochemistry. Electron transfer from aqueous iodide to water has been studied by ultrafast X-ray absorption spectroscopy (XAS)^23^ and femtosecond time-resolved fluorescence spectroscopy with polychromatic detection.^24^ Using XAS, a transition from hydrophilic solvation, with the hydrogen atoms of water pointing towards I^−^ to hydrophilic solvation where they point away from I^0^ was observed. An in-depth analysis and comparison with *ab initio* calculations revealed the formation of a transient I^0^(H_2_O) complex with a lifetime of 3–4 ps within the water cavity. Using femtosecond fluorescence spectroscopy, the initial CTTS emission was found to originate from a broad distribution of initial solvent cage configurations, leading to electron ejection times between <100 fs and 400 fs.

Section VII of this review is dedicated to inter-site charge transfer involving transition metal oxides (TMOs) (TiO_2_ and ZnO). Using picosecond and femtosecond X-ray absorption spectroscopy, charge carrier dynamics in amorphous and anatase TiO_2_ was investigated.^25,26^ It was found that 100 ps after photoexcitation, the electrons are trapped in the defect-rich surface shell of anatase TiO_2_, whereas they are trapped in the bulk of amorphous TiO_2_. Both forms of N719-dye-sensitized TiO_2_ were shown to trap the electrons in two types of traps located at the outer surface, with lifetimes ranging from nanoseconds to tens of nanoseconds. While being element-specific, and therefore substrate-specific, time-resolved X-ray absorption spectroscopy is mostly sensitive to trapped charges. A substrate-specific approach that in addition detects the free carriers consists of detecting the effect of electron injection on the excitonic transition of the metal oxide substrate upon excitation of the sensitizer. This has recently been demonstrated using ultrafast deep-ultraviolet spectroscopy.

## II. BASIC CONCEPTS

This section briefly reviews some of the basic concepts of charge migration and charge transfer. Since charge transfer on the time scale of nuclear motion and above has been extensively discussed in the literature (see, e.g., Ref. 1), here, we focus on charge migration on electronic time scales (see, e.g., Refs. 7–9 and 27–39). Starting from the simplest case of a superposition of electronic states with fixed nuclei, we discuss charge migration and the conditions under which it can take place. We subsequently introduce the role of nuclear motion and discuss how it may affect charge migration. Finally, the relation between charge migration and charge transfer is briefly discussed.

### A. Electronic superposition states

**FIG. 1.**
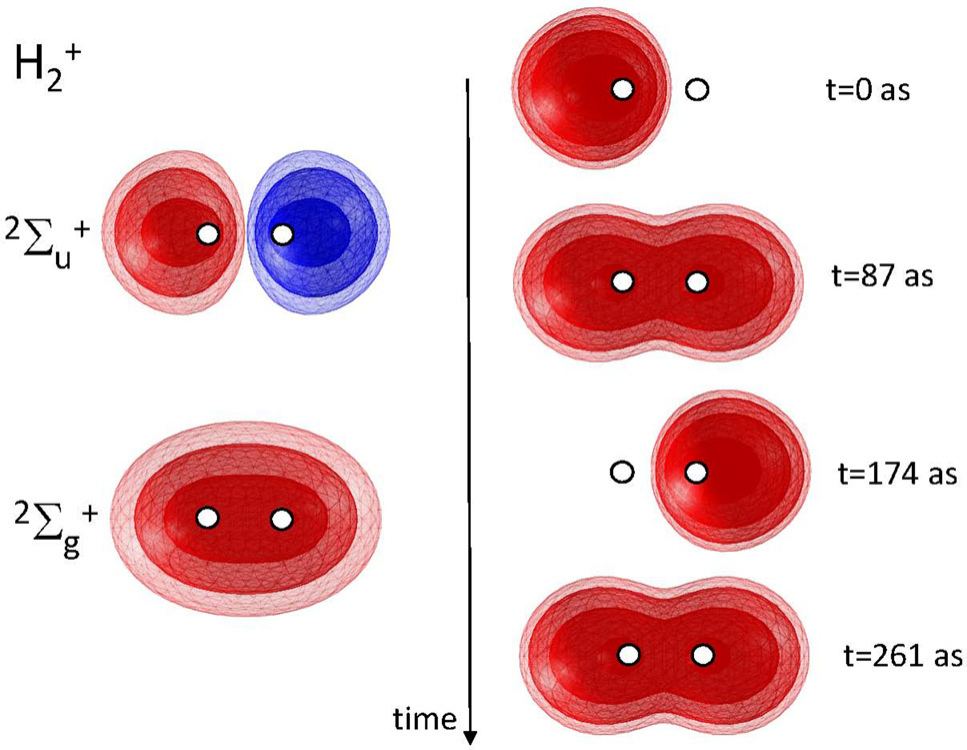
Electronic wavefunctions of the ground Σ2g+ and first electronically excited Σ2u+ states of $H_{2}^{+}$ (left) and time-dependent electron density following the preparation of the superposition state $\left(\Phi_{g}+\Phi_{u}\right)/\sqrt{2}$ at *t* = 0 (right).

The simplest situation supporting the spatial displacement of electronic charge corresponds to a coherent superposition of two electronic states, e.g., the lowest-lying Σ2g+ and Σ2u+ states of $H_{2}^{+}$. This situation is illustrated in Fig. 1. Denoting the electronic wavefunction of these two states $\phi_{g}$ and $\phi_{u}$, and assuming the nuclear positions to be fixed at the equilibrium geometry of the electronic ground state, the total electronic wavefunction of the coherent superposition state is then expressed as

$$\psi =\phi_{g}+\phi_{u}\;\text{exp}\;\left(-i\Delta Et/\hbar \right),$$ (1)

where Δ*E* is the energy separation of the two states, which amounts to 11.83967 eV at the equilibrium geometry of the electronic ground state.^40^ The time-dependent electron density is given by

$${\left|\psi \right|}^{2}={\left|\phi_{g}\right|}^{2}+{\left|\phi_{u}\right|}^{2}+2\phi_{g}\phi_{u}\;\text{cos}\;\left(\Delta Et/\hbar \right),$$ (2)

which migrates from one hydrogen atom to the other with a period of 348 as.

Coherent superposition states will thus, in general, display time-dependent electronic densities, i.e., charge migration. One prerequisite, however, is the existence of spatial overlap between the electronic wavefunctions describing the charge in each of the eigenstates. When such overlap exists, the cross term in Eq. (2) differs from zero, which leads to a time-dependent charge density. In the absence of overlap, the cross term is equal to zero and the density is time independent.

### B. Requirements for triggering charge migration

The preparation of an electronic superposition state is thus a prerequisite for observing charge migration. Since electronic dynamics typically take place on sub-femtosecond time scales, attosecond pulses are often required. However, attosecond pulses usually have photon energies lying far above the ionization threshold of most species. Therefore, experimental^7,8^ and theoretical works^9,34,36,41^ on charge migration have so far focused on the dynamics of ionized species. The remainder of this section discusses this case in more detail. We, however, note that electronic superposition states have also been created in neutral molecules using impulsive stimulated Raman scattering.^42^ The electronic dynamics of this superposition state have been followed by high-harmonic spectroscopy^42–44^ and photoelectron holography.^45^ Since both of these techniques offer sub-cycle temporal resolution,^46–50^ they will also be suitable for following charge migration in neutral molecules.^30,31,35,39,51^ Very recently, the first optical sub-femtosecond pulses have been reported and applied to prepare electronic superposition states in neutral atoms.^52^

Ionization of a multi-electron atom or molecule by a strong laser field generally populates multiple electronic states of the cation. This fact has been known since the pioneering work in 1991.^53^ This topic has played an important role in recent experiments using strong laser fields, which demonstrated the population of multiple final states using either high-harmonic spectroscopy^48,54,55^ or photoelectron-photoion spectroscopy.^56,57^ However, charge migration will only occur when a coherent superposition of electronic states is created. Experimental evidence for the creation of electronic coherence has been obtained in strong-field ionization^58–60^ and single-photon ionization.^7^ The case of strong-field ionization has been studied in the most general context in Ref. 61 and single-photon ionization in Ref. 62. The conditions for creating electronic coherence upon ionization can be summarized as follows. The photoelectrons associated with each of the ionic eigenstates must be partially indistinguishable. This condition translates into the requirement for overlap in the kinetic-energy domain and into the fact that the photoelectrons must share an irreducible representation of the appropriate symmetry group. Under these conditions, the ion is left in a coherent superposition of electronic states, which supports charge migration. These considerations apply to both single-photon and strong-field ionizations, but some subtleties remain. The requirement of overlap in the kinetic-energy domain is sufficient in the case of single-photon ionization with Fourier-transform-limited pulses, but not in the case of strong-field ionization.^61^ In the latter case, the photoelectron spectra are usually extremely broad, such that another factor becomes dominant, which is the temporal confinement of ionization. Reference 61 shows that the Fourier-transform of the instantaneous ionization rate provides a good measure of the spectral bandwidth over which strong-field ionization can prepare coherence. This “coherence window” has a typical width of ∼4 eV for ionization by an 800-nm pulse, such that charge migration with periods only longer than ∼1 fs can be initiated with a high degree of coherence by strong-field ionization with 800-nm pulses. This period increases with the wavelength of the driving field.

The most general approach for describing charge migration induced by ionization consists of using the wavefunctions of the exact electronic eigenstates of the molecular cation. On this basis, the time-dependent electronic density can be calculated. Alternatively, the density of the missing electronic charge can also be given, in at least two different ways. One possibility consists of using the difference in the total time-dependent electron densities of the ion and those of the neutral molecule before ionization. This representation has been chosen, for example, in Refs. 9 and 63–65. Alternatively, the missing charge, or electron hole, can also be represented by projecting the *N* − 1-electron wavefunction of the cation on the *N* electron wavefunction of the initial neutral state, resulting in a time-dependent one-electron wavefunction that is similar to the time-independent definition of the Dyson orbital. This approach has been chosen in Ref. 8. Comparable representations based on time-dependent linear combinations of static Dyson orbitals and a tomographic approach within a conceptual single-active-electron framework have been used in Refs. 48 and 55, respectively.

A particular type of charge migration arises when the configuration interaction among the electronic states of the cation plays a role. This situation has been the object of intense theoretical investigations (e.g., Refs. 9, 29, 32, 33, 37, and 63–65). In all of these studies, ionization was represented by the instantaneous annihilation of an electron from a canonical Hartree-Fock orbital of the neutral molecule. Since the ionic states in this situation cannot be represented by a single one-hole configuration of the neutral molecule, but generally require multiple one-hole and also two-hole-one-particle configurations,^66^ coherent superpositions of multiple electronic states are naturally created in this situation. The ensuing charge migration is, however, identical, whether or not the superposition state has been created in this particular way. Nevertheless, experiments on charge migration in configurationally mixed cations could yield interesting information on electron correlation,^67^ provided that the photoionization matrix elements can be calculated with sufficient accuracy. This situation is discussed in a recent Perspective article.^68^

### C. The role of nuclear motion

In all of these considerations, the nuclei have been assumed to be fixed. In all real molecules, different electronic states have different equilibrium geometries. When charge migration is triggered by ionization, a coherent superposition of multiple electronic states of the cation is created, triggering different nuclear wave-packet motions within each electronic state. A displacement along nuclear coordinates will modulate the vertical energy gap, resulting in different components of the nuclear wave packet (WP) displaying different charge-migration time scales [according to Eq. (1)]. This will result in a dephasing of the electronic dynamics. These effects have been studied in detail in recent theoretical works (see, e.g., Refs. 38 and 69–73).

Finally, we note that charge migration is periodic in time by definition. Permanent charge transfer, therefore, requires a mechanism of electronic dephasing or decoherence. Nuclear motion can naturally play this role, as discussed, but other sources of decoherence, such as coupling to an environment represent additional possibilities.

### D. Charge transfer

In contrast to charge migration, charge transfer designates a process in which charge relocates permanently from a “donor” to an “acceptor.” These can be different sites within a molecule, the central atom and a ligand in metal complexes, two different molecules, an atomic or a molecular solute and a solvent, or different sites with a nanoparticle, among other possibilities. All of the mentioned cases are illustrated in this review. Depending on the level of complexity and the desired accuracy, many different models have been developed to understand and predict the rates of charge-transfer processes. Since these models are well described in textbooks,^1^ we will not discuss them here. Instead, we will briefly highlight the key differences and the relationship between charge migration and charge transfer.

In the most elementary case, charge transfer occurs when the system evolves from an initial state “charge on the donor” to a final state “charge on the acceptor,” along a reaction coordinate. This evolution is usually driven by an energetic stabilization and is most frequently described by classical rate laws. The most prominent example of such descriptions is Marcus theory,^2,3,74^ which links the rate of charge transfer

$$k_{\text{CT}}=Ae^{-\frac{\Delta G^{*}}{k_{B}T}}$$ (3)

to a prefactor *A* that depends on the nature of charge transfer (unimolecular or bimolecular) and the Gibbs free energy of activation. This quantity

$$\Delta G^{*}=\frac{\lambda }{4}{\left(1+\frac{\Delta G^{0}}{\lambda }\right)}^{2}$$ (4)

can be expressed in terms of the Gibbs free energy of reaction $\Delta G^{0}$ and a reorganization term

$$\lambda =\lambda_{s}+\lambda_{v},$$ (5)

that contains a solvational ($\lambda_{s}$) and a vibrational ($\lambda_{v}$) contribution.

Marcus theory is a purely classical theory. It does not take into account any type of coherence, neither electronic, nor vibrational. It describes the rate of the overall charge-transfer process which includes electronic and structural rearrangements. Therefore, the time scale of charge transfer described by Marcus theory is limited to that of nuclear motion, i.e., several tens of femtoseconds, but in most cases, charge transfer is even significantly slower than this (up to microsecond time scales, but even much slower reactions are known). The importance of coherence in charge transfer has, however, been highlighted by several recent experiments. For example, electronic coherence was invoked in Ref. 75 to explain the quantum beats observed in charge-transfer oscillations in photosynthetic complexes. The quantum beats observed in photovoltaic blends^76^ were attributed to coupled electronic and nuclear coherences. Understanding the role of electronic or vibronic coherences in charge transfer requires a quantum-mechanical description that goes beyond the classical Marcus theory. An important open question is the role of purely electronic charge migration in such charge-transfer processes. In the limit of a sufficiently short excitation event, several electronic states will be populated in all charge-transfer systems, enabling charge migration to take place. Different vibrational dynamics taking place in these electronic states may dephase the electronic dynamics and lead to a situation where the subsequent dynamics resulting in charge transfer is controlled by nuclear motion. However, the electronic coherence could also persist for long times and control nuclear dynamics in cases where the Born-Oppenheimer approximation breaks down,^70^ e.g., at conical intersections that are ubiquitous in electronically excited states. Answering these fascinating open questions will require attosecond time-resolved experiments to be performed in relatively complex systems and will offer an exciting new frontier for physical chemistry.

## III. CHARGE MIGRATION

### A. Reconstruction and laser control of attosecond charge migration in spatially oriented molecules

Purely electronic charge migration has been predicted to take place on few-femtosecond to attosecond time scales in most molecular systems (see Refs. 37 and 77 for recent reviews). Its observation and control in real time therefore represents a considerable challenge for state-of-the-art experimental techniques. The existence of charge migration, its time scale and lifetime, as well as the possibility of controlling it in spite of its ultrafast time scale, represent key topical questions in the field of charge transfer. Recently, a quantum beat with a period of ∼4.3 fs has been observed in a specific ionic fragment in an attosecond-XUV-pump femtosecond-IR-probe experiment on the phenylalanine molecule.^7^ Frozen-nuclei TDDFT calculations showed similar dynamics when the electron density at the nitrogen atom was evaluated. On this basis and its ultrashort time scale, the observed dynamics were attributed to the electronic dynamics in the phenylalanine cation.^7^ Here, we discuss a recent work from the Wörner group,^8^ in which charge migration on a time scale below 1 fs has been resolved in space and time. In the same experiment, first evidence has been obtained that intense laser fields can be used to control charge migration on sub-femtosecond time scales, an aspect that we further detail in this review.

The capability of high-harmonic spectroscopy to resolve attosecond charge migration originates from its sensitivity to the electronic structure on one hand and its intrinsic time-to-frequency mapping on the other hand. The first aspect has been extensively studied, starting with the tomographic imaging of molecular orbitals,^78^ the interpretation of high-harmonic spectra in terms of two-centre interference,^79–81^ Cooper minima,^82,83^ giant resonances,^84^ and shape resonances.^85–89^ The second aspect was first exploited to reconstruct nuclear dynamics following strong-field ionization,^46,47^ to interpret the intensity dependence of the spectral position of an intensity minimum in CO_2_ (Ref. 48) and tomographically reconstruct electron-hole wavefunctions averaged over a duration of ∼0.6 fs in $N_{2}^{+}$.^55^ The role of laser-induced transitions in the cation has been discussed in Refs. 90 and 91.

**FIG. 2.**
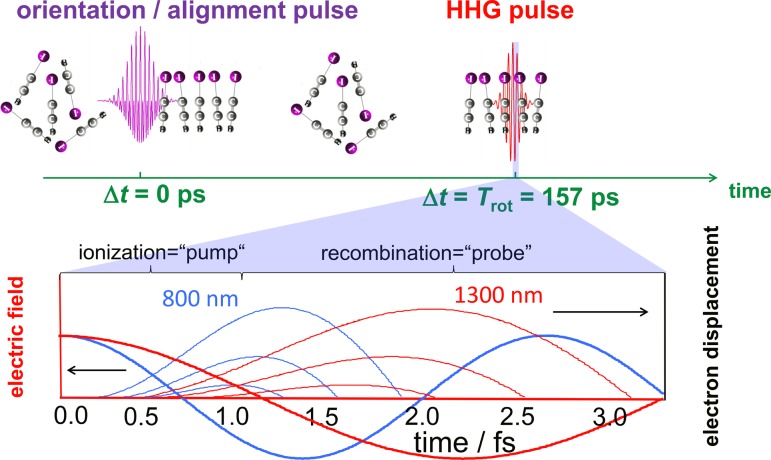
Concept of the experiment: The molecules are dynamically oriented using a two-color laser pulse. High harmonics are generated at the first full rotational period (upper panel). By selecting the short trajectories (thin lines, lower panel), a unique transit-time-to-energy mapping in the laser field (thick lines) is achieved. Changing the wavelength from 800 nm (blue) to 1300 nm (red) is equivalent to selecting a different window of excursion times for the continuum electron.

The concept of the present experiment^8^ is illustrated in Fig. 2. It relies on the detailed characterization of the high-harmonic emission from impulsively oriented molecules in the gas phase. The high-harmonic generation (HHG) pulse was delayed with respect to the orientation pulse consisting of the phase-controlled superposition of fundamental and second-harmonic laser pulses^88,92–95^ by one full rotational period of the molecule to ensure the absence of an orientation field during HHG. High-harmonic emission was studied from laser pulses with two different driving wavelengths (800 nm and 1300 nm). The lower part of Fig. 2 schematically illustrates the spatio-temporal structure of the electron trajectories driven by these two laser fields. Whereas the 800-nm field sample electron transit times are between ∼0.9 and ∼1.5 fs, the 1300-nm field accesses the window from ∼1.3 to ∼2.2 fs.

The observables in this experiment were (i) the spectral intensity and (ii) the phase of high-harmonic emission from molecules aligned parallel or perpendicular to the laser field, as well as (iii) the spectral intensity of even and odd harmonic orders for molecules oriented parallel to the driving laser field. Observable (i) was obtained by recording the high-harmonic spectra with a flat-field spectrometer and determining the ratio between the emitted intensities in the cases of parallel and perpendicular alignments. Observable (ii) was measured by a technique known as 2-source interferometry,^48,96–99^ which was extended to wavelengths beyond 800 nm for the first time. This technique consists of recording the position of the spatial interference fringes between high-harmonic emission from an aligned and a non-aligned (isotropic) ensemble of molecules. Using a half-wave plate to rotate the polarization of the alignment field in space enabled the measurement of the phase of high-harmonic emission as a function of the alignment angle. The last observable (iii) was obtained by recording the high-harmonic spectrum of oriented molecules and evaluating the ratio between the even and the two neighboring odd harmonic orders.

Even without the support of a model, the experimental data reveal the presence of electronic dynamics taking place on sub-cycle time scales. The intensity ratios of molecules aligned parallel or perpendicular to the laser field reveals a minimum that is located at 23.2 eV with a driving wavelength of 800 nm and at 35.3 eV for a driving wavelength of 1300 nm. These two very different locations of the minimum, in spite of the similarity of the applied peak intensities (1.1 and 0.9 × 10^14^ W/cm^2^, respectively), show that the electronic structure of the molecular cation has changed between the time of ionization and recombination.^100^ This wavelength dependence of the minimum is the direct consequence of the wavelength dependence of the time-to-frequency mapping in high-harmonic spectroscopy. Minima that reflect a static electronic structure, in contrast, do not depend on the intensity or wavelength of the driving field and are always located at the same position in photon energy.^81,101,102^

The reconstruction of charge migration from high-harmonic data was a challenging problem that was solved by maximizing the number and the diversity of observables and by developing a unifying theoretical model that linked these observables to the time-dependent population and relative phases of the electronic states of the molecular cation. In the case of HCCI^+^, strong-field ionization of the neutral molecule only populates the lowest two electronic states of the cation (denoted ${\tilde{X}}^{+}$ and ${\tilde{A}}^{+}$, in what follows), as verified by time-dependent density-functional-theory calculations carried out by the group of Bandrauk.^8^ Charge migration could therefore be reconstructed from the knowledge of only two populations, i.e., one fractional population considering normalization and one relative phase. The present model makes use of some of the most accurate currently available theoretical methods to link the observables to the unknown populations and phases.

The three observables (aligned-to-anti-aligned intensity ratio and phase difference, as well as even-to-odd intensity ratio) measured for each harmonic order were used to reconstruct two unknowns for each transit time, i.e., the fractional ${\tilde{X}}^{+}$-state population and the relative phase Δϕ of the two electronic states. Additionally, a global fractional population and a relative phase at the time of ionization (“t = 0”) were determined, for both parallel and perpendicular alignments. Since 3*N* observables have been measured, where *N* represents the number of harmonic orders, but only 2*N *+* *2 unknowns have been reconstructed, the problem is over-determined, which provided the opportunity to test the ability of the model to reproduce the observed data. This test was very successful (see supplementary material of Ref. 8).

Our model relies on electron-molecule quantum scattering calculations to obtain the phase and the amplitude of photorecombination matrix elements with near-quantitative accuracy (see e.g., the supplementary material of Ref. 103). Quantum-orbit theory with the saddle-point method has been used to describe the propagation of the continuum electron. This approach was validated experimentally in Ref. 104. Nuclear motion of HCCI^+^ following strong-field ionization was included through the approach outline in Ref. 105, i.e., through nuclear auto-correlation functions^46,106^ derived from experimental photoelectron spectra.^107^ Finally, since the molecules were not perfectly aligned, the variation of the strong-field-ionization rate with the alignment angle was also required. It was taken from calculations based on the weak-field asymptotic theory.^108,109^ This approach was also validated experimentally for other polar molecules.^45,110,111^

The reconstruction of charge migration makes use of the general property that the transition-dipole moment is a vectorial quantity. It lies perpendicular to the molecular axis in the case of the ${\tilde{X}}^{+}↔{\tilde{A}}^{+}$ transition of HCCI^+^. As a consequence, there is no laser-induced population transfer between the two states. Moreover, since the Stark shifts of the two states are nearly identical for molecules lying parallel to the driving field, the charge migration dynamics for perpendicular alignment can be expected to be very similar to the field-free dynamics. In contrast, molecules aligned parallel to the driving field will experience very pronounced laser-induced population transfer and very large time-dependent Stark shifts. The case of parallel alignment, therefore, provides the opportunity to study laser-controlled charge migration.

**FIG. 3.**
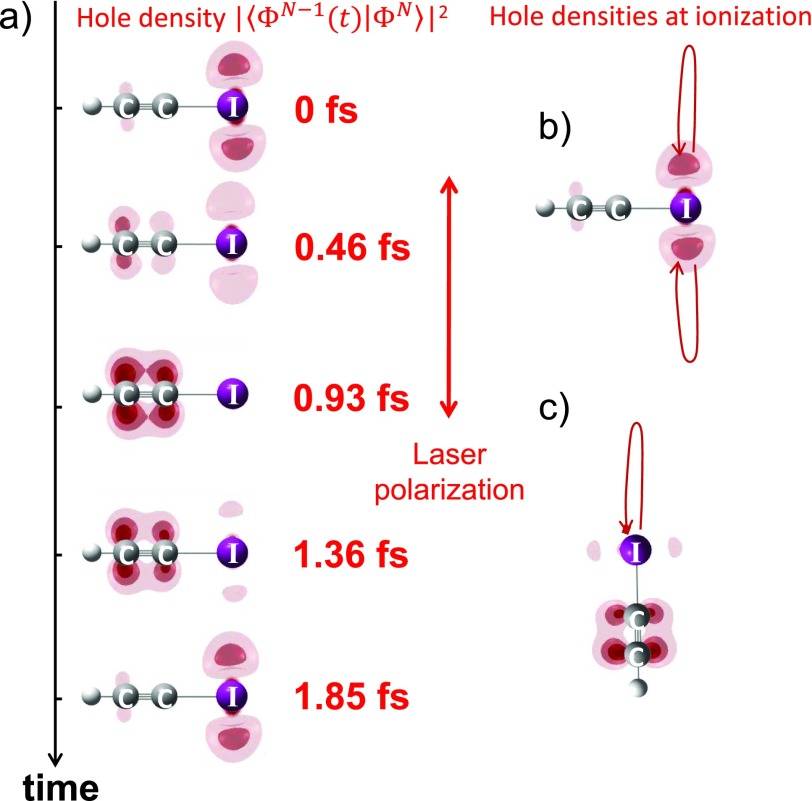
(a) The reconstructed electron-hole density is displayed as a function of time after strong-field ionization of HCCI molecules aligned perpendicular to the polarization of the driving laser pulse. The reconstructed hole densities at the time of ionization are also illustrated for molecules aligned perpendicular (b) or parallel (c) to the laser field.

The results from the reconstruction of charge migration in the quasi-field-free case are shown in Fig. 3(a) and the electron-hole densities at the time of ionization (“t = 0 fs”) are shown in Figs. 3(b) and 3(c), for perpendicular and parallel alignments, respectively. In the case of perpendicular alignment, the electron hole is created on the iodine atom. The electron hole subsequently migrates to the acetylene unit within less than 1 fs before returning to its initial position. In the case of parallel alignment, ionization from the iodine-side of the molecule dominates, but the electron hole is created on the acetylene side. This result is counter-intuitive at first sight. However, all-electron *ab-initio* calculations [CASSCF(13,11)//cc-pVTZ] with static applied electric fields confirm that the electronic ground state of HCCI^+^ in a strong electric field pointing from I to H corresponds to an electron hole located on the acetylene side. The reconstruction therefore suggests that strong-field ionization prepares the field-dressed ground state of HCCI^+^ in this situation.

**FIG. 4.**
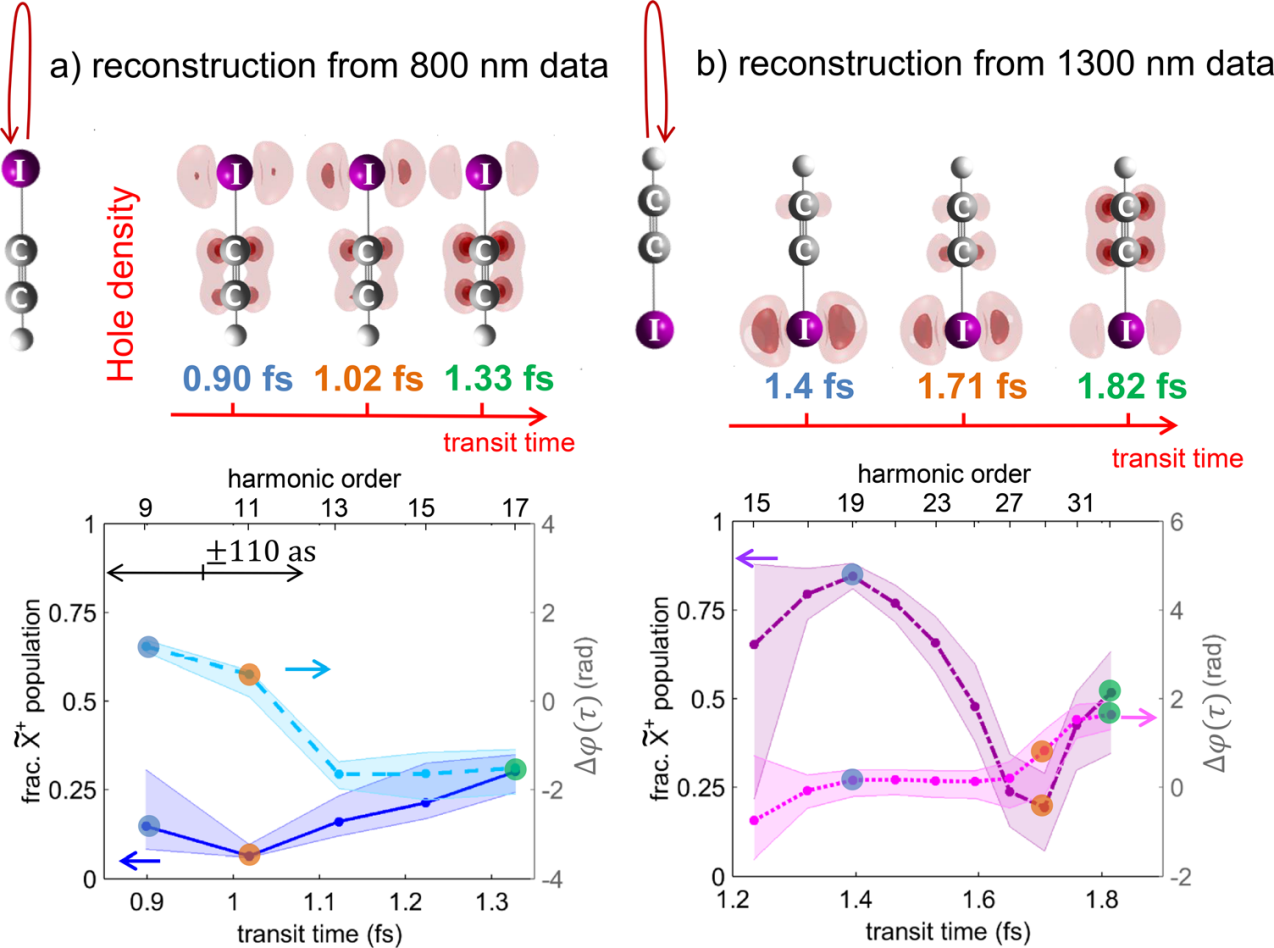
Electron-hole densities for HCCI molecules aligned parallel to the polarization of the laser field reconstructed from high-harmonic emission driven by an 800-nm (a) or 1300-nm (b) driving laser field. The lower panels show the fractional populations of the ${\tilde{X}}^{+}$ state (left vertical axis: the time-dependent population of the ${\tilde{A}}^{+}$ state is given by normalization) and the relative phase between the ${\tilde{X}}^{+}$ and ${\tilde{A}}^{+}$ states (right vertical axis). Panels (a) and (b) illustrate the dynamics following ionization from the iodine and hydrogen sides, respectively.

Figure 4 shows the results of reconstruction of laser-controlled charge migration. The top part of the figure shows snapshots of the electron-hole density at selected values of the transit time. The bottom panels show the reconstructed fractional ${\tilde{X}}^{+}$-state population and the relative phase as a function of the transit time. This reconstruction provides quantum-mechanically complete information about charge migration following ionization from either the iodine or the hydrogen side, for the time windows covered in the 800- and 1300-nm experiments. For compactness, only two out of the four sets of results are shown in Fig. 4. All results show a rapid variation of the ${\tilde{X}}^{+}$-state population over the reconstructed sub-femtosecond temporal window. The reconstruction from 800-nm data shows an almost-complete depletion of the ground-state population, followed by a partial repopulation. The relative phase shows a rapid variation around the time of maximal depletion. The 1300-nm data show a local maximum of the population, followed by a local minimum, which is again associated with a phase jump. These results are in qualitative agreement with independent calculations based on solving the time-dependent Schrödinger equation containing the 10 lowest electronic states of HCCI^+^ and all dipole-transition matrix elements. The comparison of these 10-state calculations with 2-state calculations showed that the lowest 2 electronic states of HCCI^+^ are sufficient for obtaining converged results (see supplementary material of Ref. 8). Therefore, the results of 2-state calculations are discussed here.

**FIG. 5.**
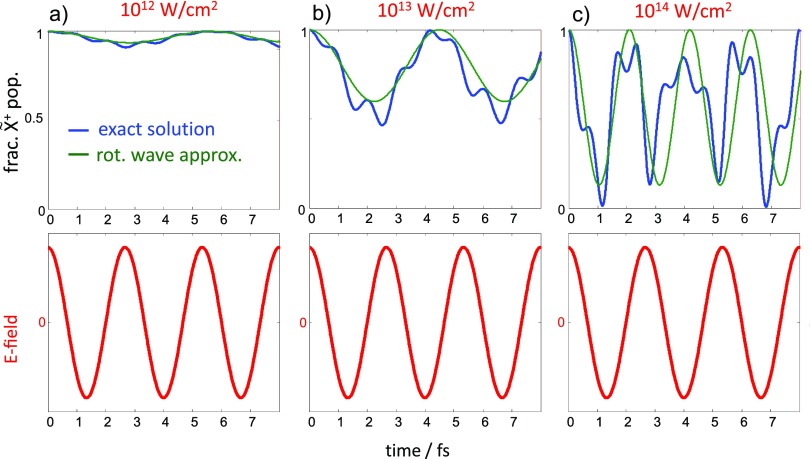
(a)–(c) Fractional population of the electronic ground state of HCCI^+^, treated as a two-level system and aligned parallel to an 800-nm laser field of the indicated intensities. The blue line shows the exact solution of the time-dependent Schrödinger equation, whereas the green line is the solution for the rotating-wave approximation neglecting higher order frequencies in the population transfer. The time-dependent laser field is illustrated in the lower panels.

Figure 5 shows the ${\tilde{X}}^{+}$-state population of HCCI^+^ exposed to 800-nm laser fields of increasing intensities given above each panel, assuming exclusive population of the ${\tilde{X}}^{+}$ state at a local field maximum, chosen to be the time of ionization. Since the laser field is non-resonant with the vertical energy-level interval of the ${\tilde{X}}^{+}$ and ${\tilde{A}}^{+}$ states [2.23 eV (Ref. 107)], relatively weak fields induce weak Rabi oscillations with periods of several femtoseconds. The rotating-wave approximation is in reasonable agreement with the exact results. With increasing intensity, the period of the oscillation decreases as expected, and the depth of the population modulation increases. At the intensity used in the present experiments (∼10^14^ W/cm^2^), the ${\tilde{X}}^{+}$ state is completely depopulated after about 1.1 fs, and the exact solution significantly deviates from the rotating-wave approximation, demonstrating the non-adiabatic character of the strongly driven electronic dynamics. In this regime, strong laser fields can therefore be used to control charge migration on the sub-femtosecond time scale.

This work has shown that (i) charge migration triggered by strong-field ionization can be reconstructed in space and time from the emission of high-order harmonics, (ii) this integrated “pump-probe” technique has sufficient temporal resolution to resolve sub-femtosecond charge migration and (iii) laser-control of charge migration on these time scales is possible. More recent theoretical results introduced the concept of a general coherence window which suggests that strong-field ionization by 800-nm fields is generally capable of triggering charge migration with periods down to ∼1 fs, increasing with longer wavelengths.^61^ The most important remaining questions concern the lifetime of the electronic coherence that drives charge migration, the interplay of charge migration and nuclear motion to reach a long-lived state with a localized charge, i.e., charge transfer, and finally the effect of this charge transfer on the subsequent chemical-reaction pathways of the molecule. These questions are likely to be a central topic of attosecond spectroscopy in the next few years.

### B. TDDFT Ehrenfest-based molecular dynamics of attosecond charge migration

Attosecond science often relies on theoretical support to fully understand and interpret the experimental outcome normally encoded in HHG spectra. An exact theoretical description of atoms and molecules in the presence of an external field requires a solution of the time-dependent Schrödinger equation; however, exact numerical solutions are only feasible for systems containing one or two electrons.^112–116^ In practice, the theoretical description of the electron dynamics in many-electron systems requires adopting approaches that neglect up to a certain degree the quantum behavior of either electrons and/or nuclei. Thus, semi-classical models like the “simple man's” or “three-step” model^117–119^ interpret the HHG emission as the consequence of the recombination of photo-ionized electrons with the parent ion after a (classical) streaking in the external laser field. They are able to accurately predict the energy cutoffs present in HHG spectra; however, they fail in the estimation of delay times in double ionization of atoms.^120^ Following this line, the widely used single active electron approximation (SAE) considers the laser ionization as involving only one electron at a time, while the remaining electrons are considered as “frozen.” The omission of multielectron effects causes a failure of SAE for describing HHG processes^121,122^ and ionization of many-electron systems.^123^ One way of overcoming these limitations is provided by TDDFT-based methods where the electronic correlation is included through an approximate exchange-correlation functional. Works on strong-field ionization^124–132^ as well as HHG spectroscopy^121,129,133–144^ have validated the accuracy of these methods in spotting some of their weaknesses also.^124,145^ In the following, one application of TDDFT Ehrenfest-based MD is used to rationalize the electronic movement involved in the HHG of oriented HCCI molecules.

**FIG. 6.**
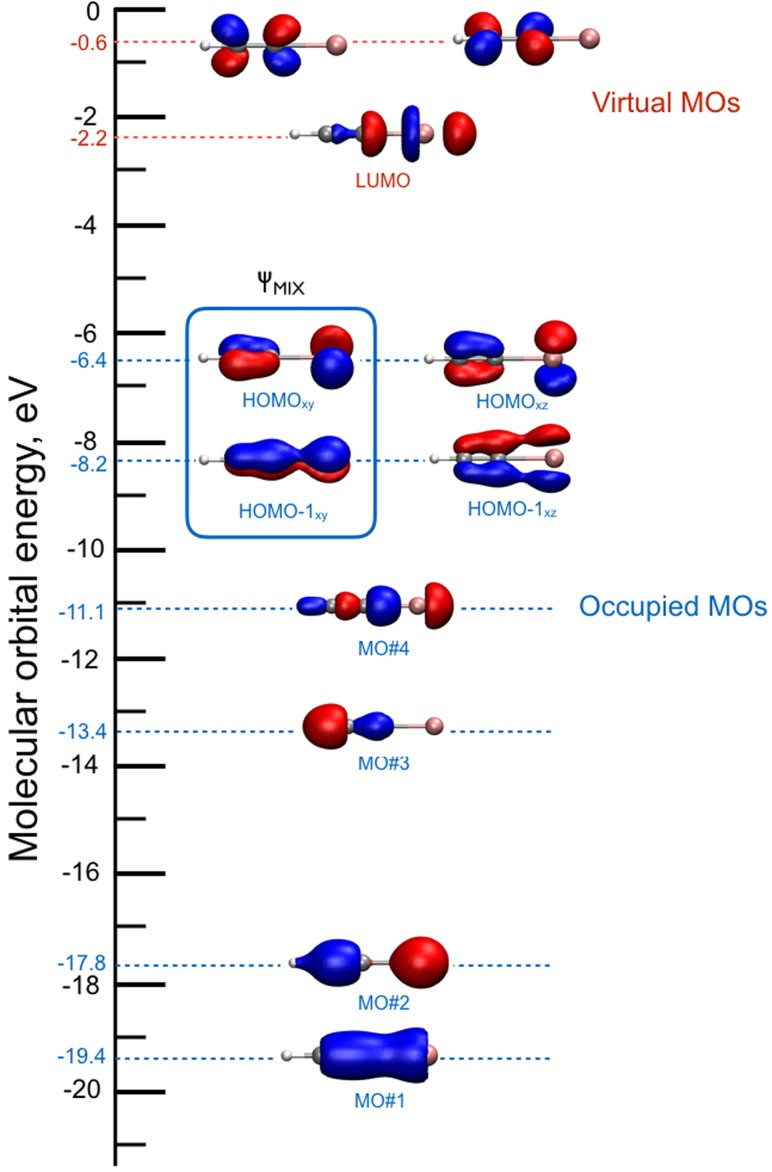
Canonical Kohn-Sham molecular orbital diagram of neutral HCCI. The orbitals involved in the superposition mixture are indicated by the square. All orbital contours are obtained using a 0.01 e/${Å}^{3}$ isovalue.

Using the iodoacetylene cation (HCCI^+^) as a test case, our intention is to assess the validity of TDDFT MD to describe the hole dynamics triggered by a “hand-made” ionization. In this sense, one picture of the electron relaxation occurring in the HCCI^+^ cation is given by the early work of Allan *et al.*^107^ The emission spectra measured show a particular band ${\tilde{A}}^{2}\Pi \to {\tilde{X}}^{2}\Pi$, at ∼2 eV. In the calculations reported here, we have prepared an electronic superposition state of the HCCI^+^ molecule by removing one electron from an orbital mixture, $\Psi_{MIX}$, of the HOMO and HOMO-1 ground-state orbitals of the neutral species (see Fig. 6)

$$\Psi_{MIX}(r,t)=\frac{1}{\sqrt{2}}(\phi_{\text{HOMO}}(r)+\phi_{\text{HOMO}-1}(r)).$$ (6)

The subsequent time-evolution of this non-equilibrium electronic distribution is simulated by means of TDDFT MD, using a cubic box of size L = 20 Å and a plane wave basis with a kinetic energy cutoff of 100 Ry. Core electrons are replaced by pseudopotentials of the standard Goedecker-Teter-Hutter form,^146^ and the exchange correlation energy is calculated using the Perdew-Burke-Ernzerhof (PBE) functional.^147^ The initial configuration of the neutral species is taken from a Car-Parrinello (CP) molecular dynamics trajectory^148^ equilibrated at 100 K using velocity rescaling. To propagate the initial perturbed density, we use the Ehrenfest-based TDDFT MD scheme as implemented in the Car-Parrinello molecular dynamics (CPMD) package,^149,150^ with an integration step of δt= 0.00024 fs.

**FIG. 7.**
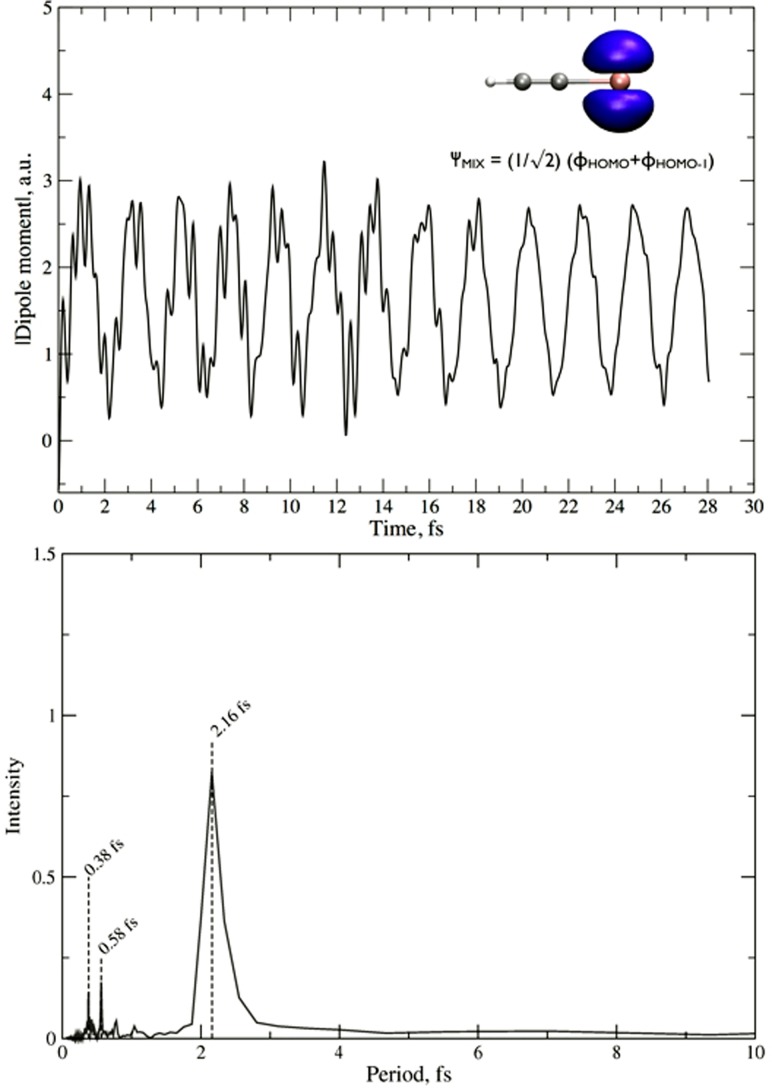
Upper panel: dipole moment oscillations of HCCI^+^ ionized from a HOMO/HOMO-1 superposition of states. In the upper right corner, the molecular orbital resulting from the linear combination is shown, $\Psi_{MIX}$, using a 0.01 e/Å^3^ isovalue. In the lower panel, the dipole moment oscillations are Fourier transformed to period-space.

The simulation is run up to 28.2 fs during which we monitor the dipole moment oscillation of the molecule caused by the relaxation of the initial excited state of the cation. This information is displayed in the upper panel of Fig. 7, together with the Fourier transformation of the oscillations (in the lower panel). One can clearly see that HCCI ionization from the HOMO/HOMO-1 superposition of states leads to characteristic charge oscillations, showing a mean feature around 2 fs that corresponds to the electronic gap experimentally observed by Allan *et al.*^107^ and also agrees well with the results of high-harmonic spectroscopy described in Sec. III A. Thus, these results validate the TDDFT EH method in the study of ultrafast dynamics triggered in the HCCI molecule, and will be compared to those obtained by simulating the neutral molecule in the presence of an intense external field.

These results confirm the existence of an extremely rapid, purely electronic charge migration in HCCI^+^ following the preparation of a superposition of electronic states, e.g., by strong-field ionization. They moreover suggest that this charge migration survives for at least 28 fs, although all nuclei were allowed to move in the present calculation. Charge migration supported by the lowest electronic states of HCCI^+^ therefore appears to be robust to decoherence induced by classical nuclear motion over several periods of vibrations. However, for a definitive assessment, the influence of the nuclear quantum nature on the electronic coherence must be investigated via quantum dynamics simulations, since it has been shown to be of particular importance for the survival of the electronic wave-packet coherence.^69–73^ Finally, the calculations also suggest that no appreciable localization of the electron-hole density occurs on the investigated time scale.

## IV. INTRAMOLECULAR CHARGE TRANSFER

### A. Charge transfer in the dissociative ionization of ethylene

**FIG. 8.**
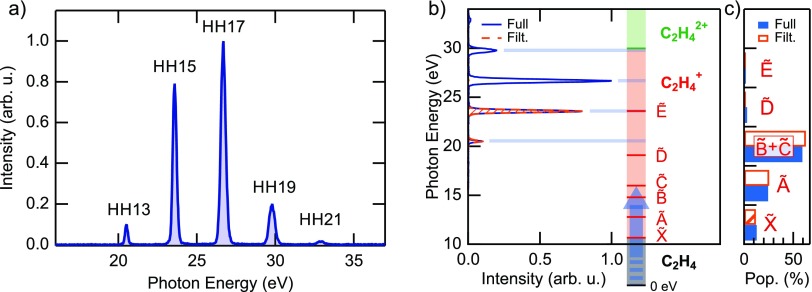
(a) Full spectrum of harmonics used as a pump in the experiment. (b) Comparison between the photon energies and the vertical excitation to the main cationic states. (c) Population of the cationic states after excitation by the spectra shown in (b), calculated with the tabulated values for monochromatic excitation.^157^ As it is possible to observe, both the full spectrum (blue) and a filtered spectrum composed of only two harmonics (orange) can efficiently populate only the first four states of the molecular cation.

After the discussion of purely electronic charge migration, we now turn to charge transfer in molecular systems and first discuss the case of dissociative ionization of ethylene. Biological molecules can often be reduced to a sequence of elementary bricks containing a carbon single, double or triple bond. The first step towards a deep comprehension of charge migration in complex molecules^7,151^ thus constitutes the study of charge dynamics in these fundamental bricks. Among them, ethylene (C_2_H_4_) represents the smallest molecule based on a carbon double bond. For this reason, its photochemistry has been the subject of intense research in the latest years.^152,153^ In Ref. 10, the Keller group studied the relaxation dynamics of ethylene after sudden ionization by an attosecond pulse train (APT) in the extreme-ultraviolet (XUV) spectral region. After ionization, the molecule is left in an excited state, which can either relax to the cation ground state or lead to molecular fragmentation. Recent experimental and theoretical studies have shown that the process of internal relaxation to the cation ground state involves mechanisms like twisting, pyramidalization and isomerization.^154–156^ This process is expected to unfold on a femtosecond time scale. Therefore, high temporal resolution is required to study the role of the conical intersections (CIs) that mediate the fast relaxation between different potential energy surfaces (PESs). The present approach is based on pump-probe measurements performed by combining an APT obtained by high-order harmonic generation in xenon with a portion of the 25-fs IR generation pulse. The XUV spectrum is composed of four main harmonics with energies between 20 and 30 eV [see Fig. 8(a)] and therefore has the energy required to ionize the molecule and populate the first excited states of the cation.^157^ In particular, a comparison with the tabulated value for the fragment yield after ionization with monochromatic light shows that the population after ionization is mainly confined to the cation ground state and its first three excited states [see Figs. 8(b) and 8(c)].

**FIG. 9.**
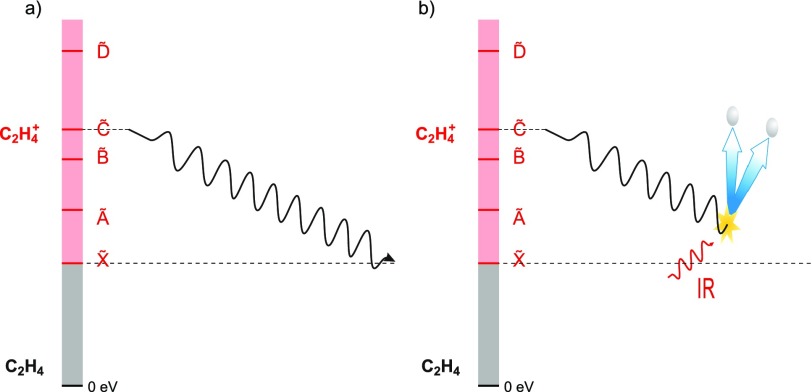
(a) Schematic drawing of the internal relaxation from an excited cation state to its ground state after XUV excitation. (b) An opportunely delayed IR pulse that can be used to stop the relaxation process by giving the molecule enough energy to lose one or two H atoms.

After the initial XUV ionization and excitation, the electronic charge will redistribute and initiate the molecular motion through which the molecule relaxes [Fig. 9(a)]. The relaxation dynamics were probed by inducing molecular fragmentation with the probe IR pulse [Fig. 9(b)] in a scheme similar to the one reported in Ref. 158.

**FIG. 10.**
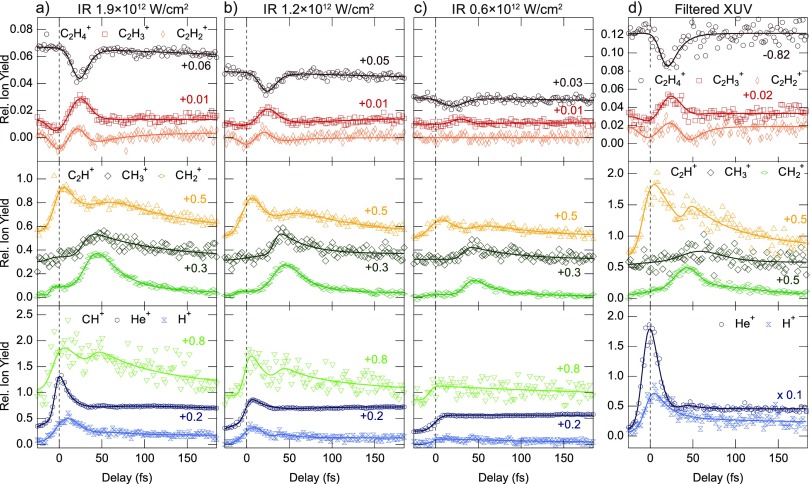
(a)–(c) IR-induced change of the ion yields for decreasing IR intensities in the probe pulse after excitation with the full harmonic spectrum of Fig. 8(a). (d) IR-induced changes for the case of excitation with the filtered spectrum composed of only two harmonics [Fig. 8(b)] and an IR intensity of 2.5 × 10^12^ W/cm^2^. The main features and their time constants are robust to the change in intensity and the XUV excitation spectrum. Adapted from the supplementary information of Ref. 10.

The pump-probe delay calibration is performed by a time-frequency analysis of the He^+^ yield obtained from the buffer gas as described in Ref. 159. In order to check the robustness of these results, different measurements with IR intensities in the range between 0.6 and 2.5 × 10^12^ W/cm^2^ were performed. Furthermore, a filtered XUV spectrum was used to exclude contamination by the dication dynamics. Figure 10 shows the experimental results of some of the different pump-probe conditions explored. All the measurements showed the same qualitative behaviour of the fragment yields. This demonstrates the robustness of the observed dynamics with respect to the IR pulse intensity and the XUV spectral content.

One possible relaxation mechanism that involves intermolecular charge transfer is isomerization. After excitation, one hydrogen atom migrates to the next carbon atom. If the molecule is fragmented in this configuration by the IR probe, it is possible to observe the generation of ${\text{CH}}_{3}^{+}$ fragments. Interestingly, all the ${\text{CH}}_{3}^{+}$ yields displayed in Fig. 10 show a clear peak around a delay of 40 fs, which corresponds to an increased probability of the isomerization. This delayed peak in the ${\text{CH}}_{3}^{+}$ yield has already been observed and related to the isomerization time of the ethylidene configuration.^155^ Thanks to the precise calibration of the zero pump-probe delay in combination with few-fs pump and probe pulses, the temporal resolution could be improved by one order of magnitude compared to the present literature. After fitting with a multi-exponential fit and taking into account the finite temporal response of the spectrometer, this allowed the isomerization time to be redefined to 30 ± 3 fs from the previous literature value of 50 ± 25 fs.^155^

The behaviour of the heavy ions (upper panels in Fig. 10) is of particular interest. The IR probe pulse induces bleaching in the $C_{2}H_{4}^{+}$ yield, which happens for delays smaller than 50 fs and with a maximum around 25 fs. The bleaching is correlated with a simultaneous relative increase of the other heavy ion yields ($C_{2}H_{3}^{+}$ and $C_{2}H_{2}^{+}$). This finding suggests that the relaxation from the excited states of the cation to its ground state happens within 50 fs. If the probe is turned off, after XUV excitation, the molecule relaxes to its ground state on its natural time scale. When the IR probe is switched on, it can interact with the excited molecule and give additional energy. If the energy transfer happens in a particular molecular geometry, the molecule can deviate from its natural relaxation path and dissociate by losing one or two H atoms. Therefore, the IR pulse can stop the relaxation process and induce a consequent bleaching of the cation yield [process sketched in Fig. 9(b)]. From the experimental results, one can conclude that it takes the molecule only ∼25 fs to reach the configuration where the probability to lose one or two H atoms after interaction with the IR probe is maximized. After ∼50 fs, the IR does not affect the $C_{2}H_{4}^{+}$ yield any longer, thus suggesting that the internal relaxation mechanism is over.

**FIG. 11.**
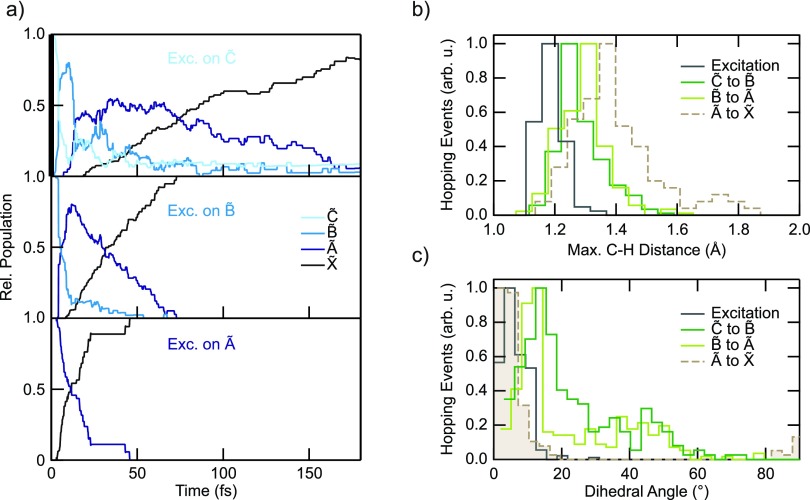
(a) Calculated population on the four lowest cationic states as a function of time after selective excitation to $\tilde{C},\;\tilde{B}$ and $\tilde{A}$ (top to bottom panels). Distribution of the C-H distances, (c), and dihedral angles, (d), of the hopping geometries for transitions between different PESs.

In order to prove this interpretation and achieve a deeper understanding on the underlying molecular dynamics, the Röthlisberger group performed calculations based on trajectory surface hopping and TDDFT. The numerical results show that after photoionization, the electron wave packet (WP) is projected onto the first four cation states from which it quickly relaxes to the ground state within 50–100 fs [Fig. 11(a)].

On one hand, this validates the interpretation of the experimental results, which therefore constitute a first experimental proof for the ultrafast time scale of internal energy conversion in the first excited states of the ethylene cation. On the other hand, the good agreement between theory and experiment allowed us to make a further step and investigate which are the physical mechanisms that form the basis of ethylene internal relaxation processes. Recent work identified two distinct classes of CIs as being responsible for the ultrafast internal conversion and relaxation, associated with planar and twisted geometries, respectively.^156^ It was proposed that the barrier between the planar and the twisted CI towards the ground state has an important impact on the branching ratio and thus the outcome of the dynamics. Previous investigations suggested, instead, that the loss of two H atoms ($C_{2}H_{2}^{+}+H_{2}$) is preceded by the formation of a bridged and ethylidene (HCCH_3_) structure^152^ leading to a CI along the C-H stretching coordinate. In order to address this problem, the distribution of C-H stretch coordinates and dihedral angles of all hopping events for transitions between different PESs [Figs. 4(b) and 4(c)] was studied. From this static representation of the PESs, efficient transitions between $\tilde{A}$ and $\tilde{X}$ are expected around three different CIs, located along the dihedral angle at 90°, along the C-H coordinate around 1.7 Å and along the planar bridging coordinate. Therefore, the present findings are consistent and in agreement with the previous theoretical studies, suggesting the existence of a CI along the C-H stretching coordinate and two $\tilde{A}$/$\tilde{X}$ CIs, one of bridged planar geometry and the other twisted.

To conclude, the improved temporal resolution of this pump-probe experiment allowed us to investigate ultrafast relaxation dynamics mediated by intramolecular charge transfer after ionization. In particular, the isomerization time in ethylene could be redefined to 30 ± 3 fs with one order of magnitude better accuracy than what was already proposed in the literature. Moreover, a detailed comparison between *a priori* calculations and the relative yield of the heavy fragments allowed us to identify the CIs as the basis of internal relaxation of the cation-excited states to their ground state, which is here proven to happen within a few tens of femtoseconds. These results thus pave the way for gaining a full understanding of the internal dynamics where precise timing of the interaction between the light and the molecule could be used to obtain optical control over the relaxation process and the associated branching ratios.

### B. Charge transfer between metal centers and ligands in metal complexes

The case of intramolecular charge transfer in transition-metal complexes is one of the most studied phenomena in coordination chemistry due to its central role in applications such as solar energy conversion and photocatalysis. Such complexes are characterized by a diversity of electronically excited states classified as metal-centred (MC), metal-to-ligand charge transfer (MLCT), ligand-to-metal charge transfer (LMCT), ligand-centred (LC), etc. One of the questions that arises is when the metal and one of the ligands form strongly mixed moieties, and electron transfer to another ligand occurs. This is the case of halogenated rhenium-carbonyl polypyridine complexes, such as [Re(X)(CO)_3_(bpy)]^1+^ (X = Cl, Br, I). According to DFT calculations,^160^ the HOMO and HOMO-1 of the halide complexes are of Re-L*π*-antibonding in character. The d*π*(Re) contribution to these orbitals decreases from ∼50% to ∼30% upon substituting the chloride ligand for iodide. The p*π*(halide) contribution concomitantly increases from ∼20% to ∼56%.^160^ The lowest unoccupied orbitals, which are of importance for the population of long-lived and inherently stable charge-transfer (CT) states under near-UV or visible irradiation, consist of several predominantly (at least 90%) π*(bpy) and higher in energy, π*(CO) levels. Therefore, the low-lying excited states for these complexes are expected to exhibit a Re → bpy and X → bpy CT character,^161^ due to the strongly mixed metal-halogen moiety of the HOMO, and this has led them to being labeled as metal-ligand-to-ligand charge-transfer (MLLCT) states. However, direct experimental evidence of this two-center electron transfer has been lacking so far. The Chergui group carried out a time resolved XAS study at the Re L_3_- and Br K-edges of the complex [ReBr(CO)_3_(bpy)] in dimethylformamide.^162,163^ They found a clear-cut signature of the electron charge leaving both the Re and Br centres, while parallel optical domain experiments had clearly established that the bpy ligand becomes reduced.^164,165^ X-ray experiments were carried out with 70 ps resolution, and the question whether the electron charge first leaves one of the centres and the hole is redistributed between the two centres still needs to be addressed. For this purpose, a significantly higher time resolution would be needed, probably in the attosecond regime.

### C. Solvation-driven charge transfer in metal complexes

**FIG. 12.**
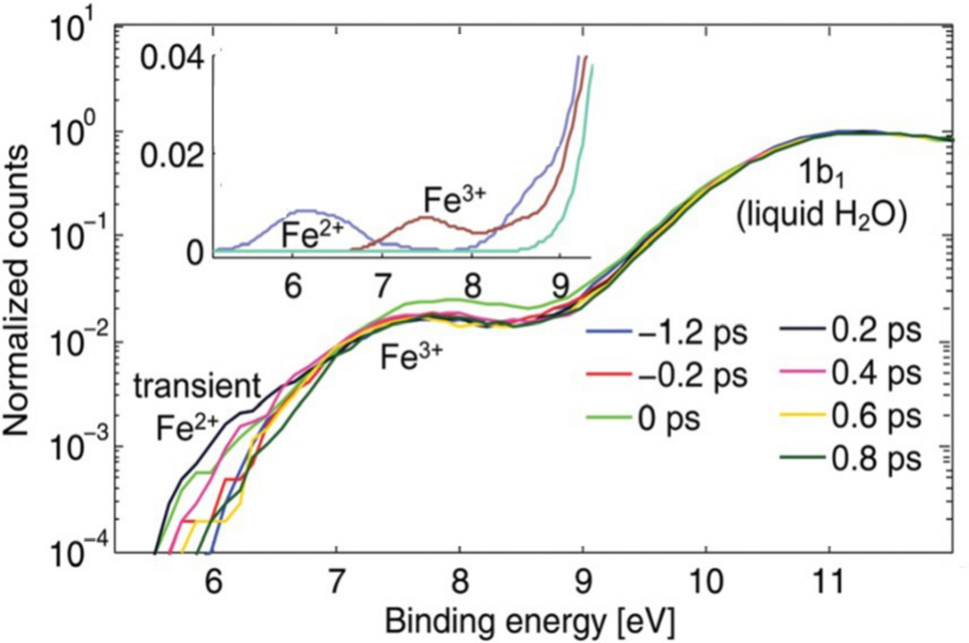
Time-resolved photoelectron spectra of aqueous ferricyanide upon photoexcitation of the LMCT band centred at 420 nm. The inset shows the static (unpumped) photoelectron spectra of water and aqueous ferro- and ferri-cyanide solutions in the region of the Fe^2+∕3+^ HOMO, easily distinguishing the oxidation state. Reproduced with permission from Ojeda *et al.*, Phys. Chem. Chem. Phys. **19**, 17052 (2017). Copyright 2017 The Royal Society of Chemistry and the PCCP Owner Societies.^12^

In the study of ultrafast intramolecular charge transfer dynamics, the recently implemented ultrafast photoelectron spectroscopy (PES) of liquid solutions is opening a new perspective, thanks to its ability to clearly identify the oxidation state of specific centres in the molecules and the absolute energy of the involved states.^11,166,167^ Within the NCCR-MUST, a source of high-harmonic generation (HHG) vacuum-ultraviolet (HHG) radiation was built and coupled to a chamber for the ultrafast PES of solutions.^168–170^ An ideal model system for intramolecular charge transfer is the ferric [Fe^III^ (CN)_6_]^3−^ whose absorption in the 400 nm region is due to a ligand-to-metal charge transfer state, and therefore its excitation leads to a reduction of the Fe centre. Since the steady-state PES spectra of both the ferrous and ferric forms of [Fe(CN)_6_] are known (inset to Fig. 12), they provide a very good basis for the analysis of the results. Figure 12 shows the PES spectra at different time delays after excitation at 400 nm. The spectra are normalised to the solvent bands, namely the 1b_1_ peak of liquid water. On the low energy side of the PES spectra at early time delays, one can clearly distinguish a new feature, whose position corresponds to that of the ferrous complex (see inset). A time scan of this feature shows that the CT is prompt, within our time resolution of 80 fs, and the newly formed ferrous species recovers in ∼500 fs in both normal and heavy water. Quite remarkably, in ethylene glycol, the decay of the ferrous centre is much longer (∼1.2 ps) pointing to a solvent effect on the back electron transfer. The fact that the latter is indeed a back ET is confirmed by transient infrared (IR) spectroscopy, which was implemented to complement the PES results.

**FIG. 13.**
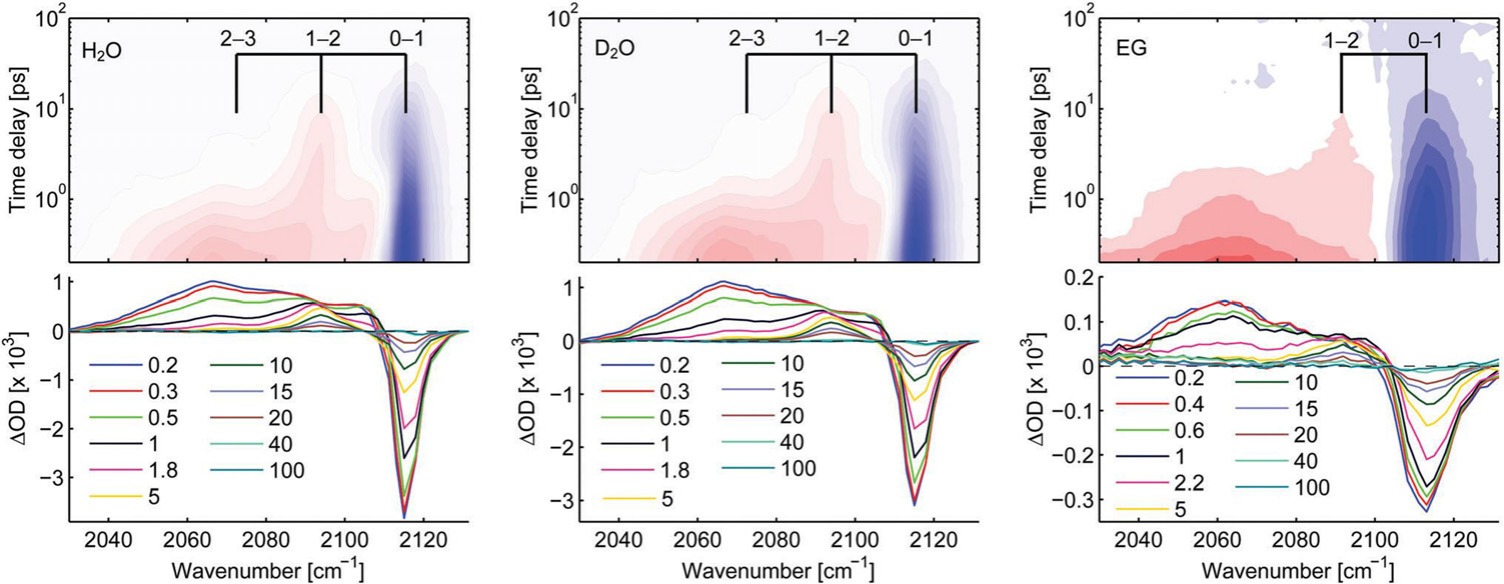
Transient IR spectra upon 400 nm excitation of [Fe(CN)_6_]^3+^ in H_2_O (left), D_2_O (centre) and ethylene glycol (right). The spacings of the grids in the 2D representations correspond to various frequencies of the T_1u_ CN-stretch mode in the electronic ground state. Reproduced with permission from Ojeda *et al.*, Phys. Chem. Chem. Phys. **19**, 17052 (2017). Copyright 2017 The Royal Society of Chemistry and the PCCP Owner Societies.^12^

**FIG. 14.**
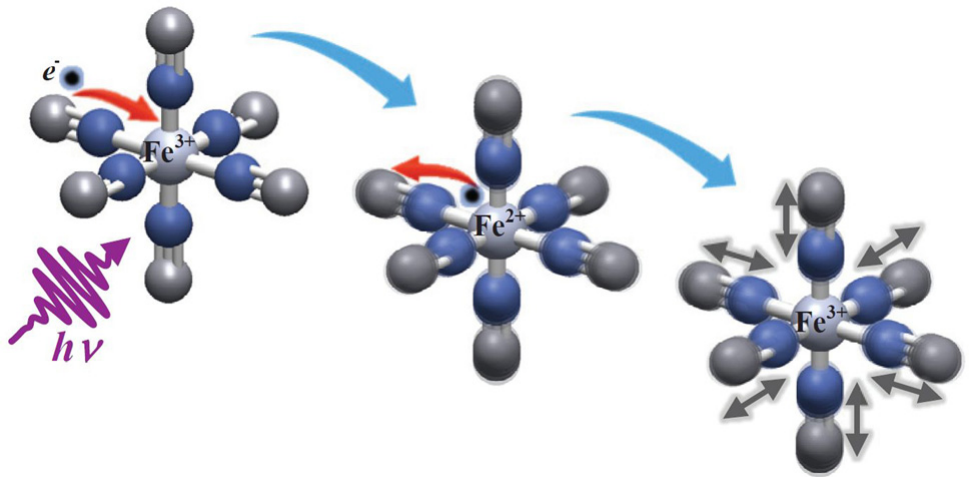
Schematic representation of the dynamics induced by LMCT excitation of ferric hexacyanide in solution. The sudden electron transfer to the metal triggers vibrational excitation of the CN ligands. The back electron transfer occurs in 500 fs leaving the molecule in a vibrationally hot state, with levels up to *v *=* *2 being populated. See Ref. 12 for details.

The transient IR spectra in H_2_O, D_2_O and ethylene glycol (EtGly) are shown in Fig. 13, and they show an immediate bleach of the fundamental CN-stretch band at 2115 cm^−1^ and the appearance of a red-shifted, broad absorption band centred at 2065 cm^−1^. It decays on a similar time scale as the Fe^2+^ signal of the PES (Fig. 12) in H_2_O, D_2_O and EtGly. This feature corresponds to the molecule with a reduced Fe^2+^ centre. Thereafter, the spectra consist of sharper positive IR bands in the 2040–2100 cm^−1^ range, which turn out to be vibrationally excited IR bands from the *v *=* *1 and 2 levels as well as a anharmonically shifted *v *=* *0 band. The vibrationally excited IR bands then decay on time scales of 1 and 10 ps, which are those measured separately in IR-pump/IR-probe experiments. The detailed analysis of these results is presented in Ref. 12, and is pictorially shown in Fig. 14. One of the main findings is that the electron transfer process triggers vibrational excitation of the CN stretch modes, due to the sudden change of the field of forces in switching from Fe^3+^ to Fe^2+^.

The origin of the much slower back ET compared to the forward one is unclear, but it may point to a role of the vibrational excitation of CN modes. This relates to the effects of CN vibrational excitation on the ET observed in molecular assemblies in the work of Khalil and co-workers^171^ and Weinstein and co-workers.^172^

Alternatively, the solvent always assists the formation of charge separation states by stabilizing the energy of the localized charges. A deep understanding of the solvation mechanisms and timescales is therefore essential for a correct description of any photochemical process in the dense phase and for designing molecular devices based on photosensitizers with CT excited states. In the last two decades, with the advent of ultrafast time-resolved spectroscopy, microscopic models describing the relevant case of polar solvation (where both the solvent and the solute molecules have a permanent electric dipole and the mutual interaction is mainly dipole-dipole) have dramatically progressed. Regardless of the details of each model, they all assume that the effect of the electrostatic fields of the solvent molecules on the internal electronic dynamics of the solute is perturbative, and that the solvent-solute coupling is mainly an electrostatic interaction between the constant permanent dipoles of the solute and the solvent molecules. This well-established picture has been proven to quantitatively rationalize the spectroscopic effects of environmental and electronic dynamics (time-resolved Stokes-shifts, inhomogeneous broadening, etc.). However, recent computational and experimental studies, including those of the Cannizzo group,^13^ have shown that further improvement is required. Indeed, in the last few years, the Cannizzo group has investigated several molecular complexes exhibiting photo-excited CT states and has found that the current description of the formation and stabilization of CT states in an important group of molecules such as transition-metal complexes is inaccurate. In particular, they showed that the solvent molecules are not just spectators of intramolecular electron density redistributions, but significantly modulate it (see Ref. 13 and references therein). These results solicit a further development of quantum-mechanical computational methods to treat the solute and (at least) the closest solvent molecules including the non-perturbative treatment of the effects of local electrostatics and direct solvent-solute interactions to describe the dynamical changes in the solute excited states during the solvent response.

### D. Solvent dynamics following oxidation reactions in model systems

Solvent molecules around metal complexes are of fundamental and practical relevance, in both catalysis and solar energy conversion.^173–175^ However, directly observing and characterizing the influence and role of the solvent is difficult experimentally mainly because of the transient nature of the process and the finite time resolution of the experiments.

A pivotal system to study solvent reorganization following photoexcitation (oxidation) is Fe-tris-bipyridine [Fe(bpy)_3_]^2+^. Iron-containing complexes constitute an important and versatile class of metal complexes. Depending on the strength of the ligands, they can exist either in a low-spin (LS) or in a high-spin (HS) state. Specifically, the [Fe(II)(bpy)_3_] complex exhibits two spin states: a LS singlet (^1^A_1_) and a HS quintet (^5^T_2_).^176,177^ Between these two states, excited-state charge transfer and spin dynamics take place (spin crossover—SCO—dynamics), which can be induced by illumination, temperature or pressure changes.^178^

Photoexcitation of the iron-complex leads to a non-equilibrium preparation of the system from where it relaxes towards a new stationary state. Such processes can be investigated with state-of-the-art X-ray spectroscopic techniques.^179^ Recently, even the response of the solvent has been probed by fs X-ray scattering and correlated to the fs X-ray emission signals.^180^

Atomistic simulations with validated force fields constitute a complement to such experiments and provide a detailed molecular-level insight into the motions and time scales involved. For the present situation, the VALBOND force field provides the necessary accuracy. It is based on valence-bond theory and is capable of more realistically describing angle bending in metal complexes.^181–183^ Unlike the simple harmonic approximation, VALBOND bending functions capture the energetics at very large angular distortions and support hypervalent compounds by means of 3-center-4-electron bonds.^182^

The system considered is one [Fe(bpy)_3_]^2+^ complex solvated in explicit water.^14^ Suitable force fields were parametrized and validated for three states: Fe(II)_LS_, Fe(II)_HS_, and Fe(III). The actual simulations were started in the Fe(II)_LS_ state, and electronic excitation to Fe(III) was induced by instantaneously changing the force-field parameters between the two oxidation states. Similarly, the relaxation process of Fe(III) to Fe(II)_HS_ was carried out by changing the force field parameters from Fe(III) to Fe(II)_HS_. Such perturbations lead to a non-equilibrium situation from which the system relaxes towards an equilibrium state.

The analysis of the molecular-dynamics trajectories included the radial distribution functions *g*(*r*) between the complex and the solvent water and the lifetime of the water molecules close to the complex. It was found^14^ that the degree of solvation decreases on a sub-picosecond time scale and originates from the excitation to the ^1,3^MLCT band. Hence, water expulsion occurs between [Fe(II)_LS_ (bpy)_3_] and the ^1,3^MLCT state. The process is electronically driven and occurs on a sub-picosecond time scale. Furthermore, it was found that relaxation of the non-equilibrium ensemble of the ^1,3^MLCT state to the equilibrium [Fe(II)_HS_ (bpy)_3_] state occurs on the picosecond time scale, which agrees with recent experiments^184,185^ and the water exchange dynamics in the inner shells close to the metal center take place on the picosecond time scale.

## V. INTERMOLECULAR CHARGE TRANSFER

### A. Long-lived charge-separated state with two electrons on the acceptor

After the discussion of intramolecular charge transfer, we now turn to intermolecular charge transfer in more complex molecules and discuss the transfer of two units of charge, resulting in a long-lived charge-separated state. Natural photosynthesis uses a photoinduced electron transfer as the primary process to convert light energy into chemical energy,^186^ and huge efforts are currently going on to mimic that process in artificial molecular systems.^187–191^ Photoinduced electron transfer in molecular assemblies has been investigated for several decades, but the vast majority of prior studies have reported exclusively on the transfer of single electrons.^192–194^ However, since the resulting radicals would be very reactive, Nature tries to avoid them as much as possible, and any useful reaction will require pairs of electrons. In particular, when considering water splitting, which is thought to be one possible solution to the energy needs of mankind, the evolution of molecular hydrogen requires the transfer of two electrons and that of molecular oxygen, an undesired but unavoidable side reaction, four electrons. Only a handful of works have appeared in the literature that report on the accumulation of two electrons at a single site in an artificially designed molecular system without the use of sacrificial reagents.^195–198^

**FIG. 15.**
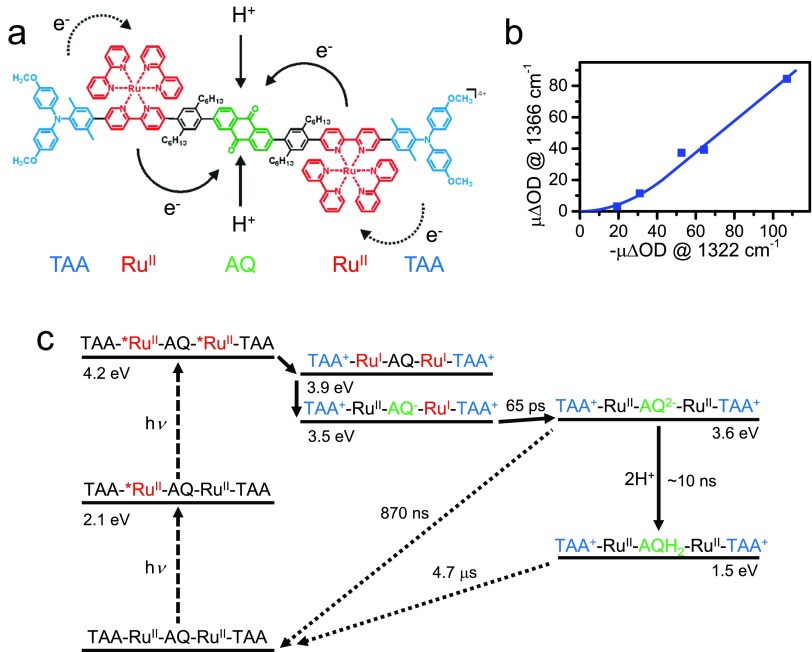
(a) The molecular assembly used to accumulate two electrons in the central anthraquinone (AQ) in a light-driven process. The electron and proton transfer steps are indicated. (b) Verification of the quadratic power dependence of the doubly reduced state AQ^2–^. To that end, the amplitude of an IR band indicative of the doubly reduced state AQ^2−^ at 1366 cm^−1^ is plotted against the bleach signal of AQ at 1322 cm^−1^. (c) Electron pathway of the doubly reduced state in an energy level scheme, with the time-scales of various electron and proton transfer steps indicated. The pathway with a single excitation leading to the singly reduced state is omitted for clarity. Adapted from Refs. 15 and 16.

In Refs. 15 and 16, a molecular assembly has been constructed exactly for this purpose, consisting of five components: two electron donors (triarylamine TAA), two $\text{Ru}{\left(\text{bpy}\right)}_{3}^{2+}$ complexes as a photosensitizer, and a central anthraquinone (AQ) as an electron acceptor, see Fig. 15(a). That pentad (as well as the triad consisting only of one electron transfer branch) has been studied by transient IR and transient UV/Vis spectroscopy, both being complementary and sensitive to different transient species in the overall reaction cycle. Upon pumping the system with a short laser pulse, the singly reduced (AQ^−^) and doubly reduced AQ^2−^ species are formed on a few 10's of picosecond time-scale, as determined from the characteristic marker-modes in the IR spectrum. As an additional evidence that the doubly reduced state is indeed formed, which has been a critical point in this work, Fig. 15(b) shows the laser-power dependence of the amplitude of the AQ^2−^ marker mode plotted against the bleach signal of an AQ marker mode. The initial quadratic power dependence proves that two photons are indeed required for AQ^2−^ to be formed. In a de-aerated acetonitrile solution, the charge-separated state lives for 870 ns, before charge-recombination to TAA occurs [Fig. 15(c)].^15^

Natural photosynthesis utilizes proton-coupled electron transfer (PCET) instead of the electron transfer discussed for the most part in this review article. In this way, “reduction equivalents” can be accumulated without actually accumulating charges. Accumulating charges would be very energy-inefficient, since the energy needed to add a new charge to an already charged compound would increase with each individual charging step, which is incompatible with the fact that the energy amount provided by a given photosensitizer is determined by its HOMO-LUMO gap, and as such is essentially a constant. Neutralizing the charge of an electron transfer with an additional proton renders the energetics of each individual transfer step more or less constant. This is, in particular, relevant when four redox equivalents have to be transferred, such as in the Mn_4_Ca-cluster used by Nature for oxygen-evolution.^186^

In order to mimic that aspect of photosynthesis also with the pentad model system, an acid has been added to the solution in a second set of experiments.^16^ In that case, a diffusion-controlled protonation step is observed after the reduction of AQ on a ∼10 ns timescale (depending on proton concentration); hence, electron and proton transfer steps are sequential in the forward reaction. Protonation stabilizes the charge-separated state significantly in terms of its energetics [Fig. 15(c)], but somewhat surprisingly, not so much in terms of its lifetime, which is prolonged only by a modest factor of ∼5. It seems plausible that AQ^2−^ in neat acetonitrile benefits from the inverted driving-force effect, since the energy gap to the charge-recombined state is quite large [3.6 eV, see Fig. 15(c)]. On the other hand, AQH_2_, which is less in the inverted regime, most likely recombines via a concerted PCET, as evidenced by a kinetic isotope effect;^16^ hence, it does not have to climb the energy barrier of AQ^2−^ for a charge recombination to occur.

To conclude this section, artificial photosynthesis will invariably rely on multi-electron redox reactions, just as natural photosynthesis does. It is therefore important to elucidate the basic principles of photodriven accumulation and temporary storage of redox equivalents, and the model system described here is an important proof-of-concept in this regard.

### B. Charge transfer in proteins

Charge (both electron and proton) transfer in proteins is an essential process of their function. Simple electron transferases include flavodoxins, blue-copper proteins, iron-sulfur proteins, and cytochromes.^199,200^ The electron-localizing groups in protein electron transfer pathways fall in an energy window of about 2 eV.^201^ Tyrosines and tryptophan amino-acid residues are involved in electron-transfer processes as intermediates. However, the redox window does not sufficiently oxidize or reduce to react with the peptide backbone and other side chains. In a way, this allows approximating proteins as wide gap semiconductors with “dopants” (redox active species) that provide electron localizing sites. *A fortiori*, in the presence of a photon, these electron transfer processes can be accelerated or new ones may be triggered. Myoglobins (Mb) represent one such class as the Chergui group recently demonstrated. Mb belongs to the family of heme proteins and consists of 153 amino acids and an active iron porphyrin (heme) centre. It contains two tryptophan residues: Trp7 and Trp14, which are located in the *α*-helix A with respective distances of 21.2 Å and 15.2 Å (centre to centre) to the heme group. In both the ferrous (Fe^2+^) and ferric states (Fe^3+^), the metal atom allows for binding a variety of diatomic ligands (including O_2_, CO, NO, and CN), which determine its function. Photoexcitation of the Trp's in Mb leads to its fluorescence, with much shortened decay times compared to the isolated case (ca. 3 ns): ∼20 ps for Trp14 and ∼120 for Trp7.^202–204^ This shortening has been attributed to FRET to the heme.^204^

**FIG. 16.**
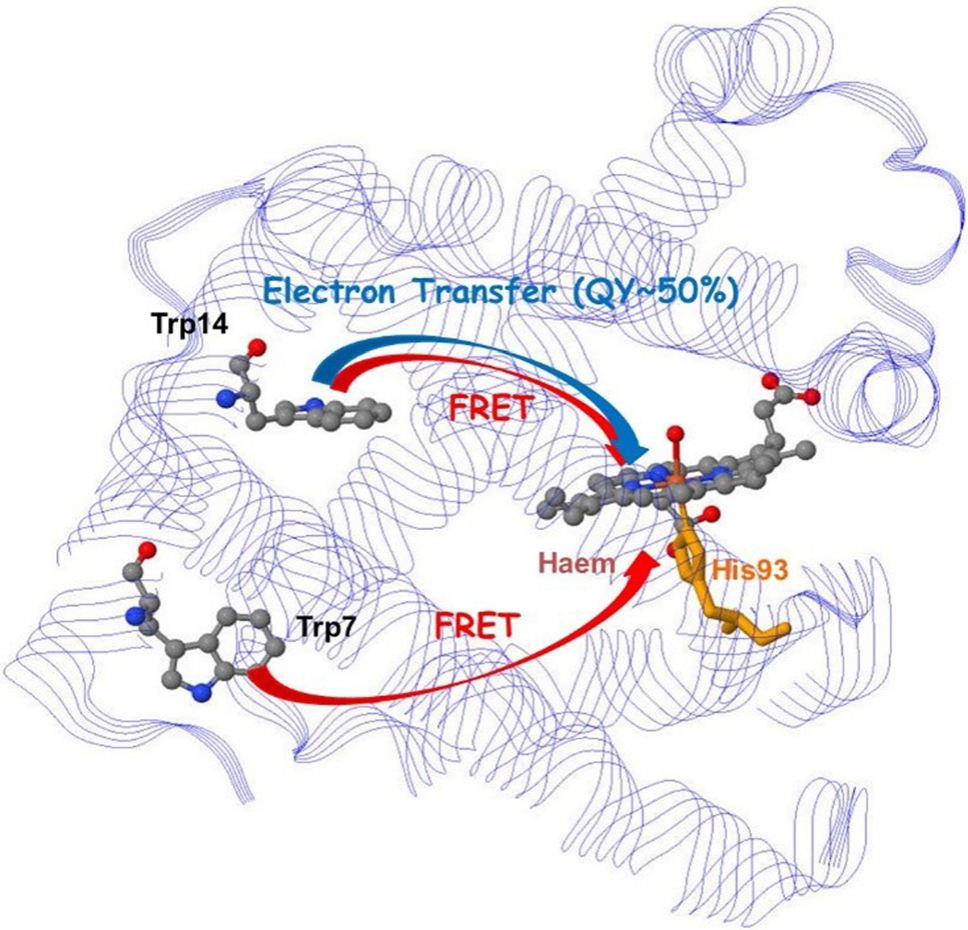
Schematic representation of the electron transfer and FRET processes between the two tryptophans (Trp) in myoglobin and porphyrin.

Using 2D deep-UV TA spectroscopy, the Chergui group revealed that in ferric myoglobins, MbCN and metMb,^17^ Trp14 undergoes a partial electron transfer to the heme, while relaxation of the more distant Trp7 is due to FRET to the heme (Fig. 16). The electron transferred from Trp14 ends up on the iron atom forming a ferrous porphyrin as was further established by UV pump/visible probe TA spectroscopy.^17^ Because the Trp fluorescence lifetimes are rather invariant to the oxidation state of the iron atom and to the presence of a ligand or its nature,^202,204^ it was concluded that the decay mechanism by electron transfer is present in all myoglobins. The Chergui group demonstrated this to be the case in the ferrous (unligated) deoxy form,^18^ this time leading to the formation of a low-valence porphyrin anion radical. A more recent work on ligated ferrous Mbs reveals the same pattern of Trp-to-heme electron transfer. In Ref. 18, it was suggested that the pathway for the electron transfer proceeds via leucine 69 (Leu69) and valine 68 (Val68) residues, which are in van der Waals contact with each other, while Leu69 is in van der Waals contact with Trp14. This was recently supported by a theoretical modelling of the Trp-heme electron transfer.^205^ While the above results of a Trp-mediated electron transfer competing with FRET are especially important for the fundamental understanding of electron transfer in biological systems, from a practical point of view, they underscore a limitation of Trp as the “spectroscopic ruler” in FRET studies of protein dynamics.^206^ Indeed, unambiguous evidence of FRET requires not only the decay time of the donor fluorescence, but also the detection of the acceptor population growth on a similar time scale, a criterion that is often overlooked in studies of protein dynamics.

### C. Charge transfer in donor-acceptor-type conjugated materials

In organic solar cells, conjugated polymers are typically blended with fullerene derivatives to form a solid-state film of 100 nm thickness, where charges are photogenerated by exciton dissociation between the two materials. To increase the efficiency of such devices, homopolymers consisting of similar repeat units are more and more being replaced by alternating donor-acceptor copolymers,^207,208^ which also show ambipolar charge transport.^209–211^ Their large repeat unit consists of an electron-rich moiety (D) and an electron-poor moiety (A). Hybridization of the molecular orbitals from D and A leads to a reduced optical bandgap, allowing better harvesting of the near-infrared part of the solar spectrum. From a photophysical point of view, this approach raises questions about the existence of dipole moments in the ground and excited states. Ideally, the excited state of conjugated polymers should be delocalized, so that the effect of localization due to (partial) intramolecular charge transfer (ICT) between the D and A moieties needs to be evaluated.

A polymer called PCDTBT {poly[N-9-hepta-decanyl-2,7-carbazole-alt-5,5‐(4,7-di-2-thienyl-2,1,3-benzothiadiazole)]} has attracted attention in 2009 as the first D-A copolymer with high photovoltaic efficiency.^212^ Scholes *et al.* have investigated the excited state evolution of dissolved PCDTBT on the <1 ps time scale using two-dimensional electronic spectroscopy (2D-ES) with high time resolution.^213^ They observed an increase of the dipole moment during relaxation, i.e., a < 200 fs increase of ICT character. To further understand the excited-state behavior of this polymer, the oligomer of PCDTBT consisting of just one D-A repeat unit was investigated (we will refer to this as CDTBT).^19,214^ Unlike the polymer, CDTBT is soluble in most organic solvents allowing for solvatochromic studies, and relaxation is slowed down in a reduced density of states compared to the polymer. Moreover, the transitions of the oligomer could be directly computed by TD-DFT at the M062X/6‐31+G* level of theory following gas phase geometry optimization in the ground and excited states.^19^ The calculations indicated an increase of ICT during vertical absorption. Nevertheless, the absorption band showed negligible solvatochromism, while the emission band strongly varied with solvent polarity, pointing to a further increase of ICT during excited-state relaxation. The partial charge transfer after relaxation in polar solvents was confirmed by a lower emission quantum yield compared to non-polar solvents.

The charge transfer relaxation in CDTBT was directly visualized using femtosecond transient absorption (TA) spectroscopy.^19^ In non-polar decane, there was practically no evolution of the TA features with time. However, in more polar solvents, strong spectral dynamics occurred with several time constants that were related to the viscosity of the solvent and followed, in particular, the reported solvation time for the investigated solvents.^215^ Therefore, solvent rearrangements seem to dominate the charge transfer relaxation, although torsional relaxation (planarization) cannot be excluded. The TD-DFT calculations indeed showed planarization of the molecule in the emitting state. In summary, a progressive increase of charge transfer character from the initially moderately polar excited state in CDTBT is mainly driven by solvent reorganization and some torsional rearrangements. Very interestingly, the Banerji group observed similar relaxation in the solid state of CDTBT (without solvent) and could even detect the presence of fully separated charges. The negative impact of excited-state localization due to ICT on the charge generation and transport in polymer:fullerene blends might therefore be counterbalanced by a positive effect of the D-A character on the exciton dissociation.

In the framework of dye-sensitized solar cell (DSSC) development, the donor-π-bridge-acceptor architecture (D-π-A) is found to be the most efficient so far. These organic dyes possess high extinction coefficients, compared to their Ru-based counterparts. Moreover, their spectral properties can easily be tuned by standard synthetic methods. Typical donors are based on coumarin, indoline, tetrahydroquinoline, triarylamine, heteroanthracene and carbazole. A substituent with the dual role of an acceptor and an anchor is commonly a cyanoacrylic acid, but can be substituted with a carboxylic acid, benzoic acid, alcohol or cyano group. The Moser group studied the dependence of the charge-carrier dynamics on the donor-acceptor distance, the π-conjugation length and the coupling of the dye with TiO_2_, by performing systematic structural and therefore electronic modification to different series of dyes. Ultrafast pump-probe spectroscopy is used as a tool for monitoring charge transfer within the molecule and into oxide substrates, and is systematically backed by theoretical modelling using DFT calculations. The impact of the π-spacer is found to inhibit back electron transfer when the conjugation is interrupted in the oxidized form of the dye.^216^ The molecular-wire role of the spacer units was highlighted by extracting an attenuation factor beta ranging between 0.04 and 0.07 Å^−1^, therefore revealing a very small dependence on the distance, which indicates an efficient conjugation between the donor and acceptor extremities of the dye. Another modification of the π-bridge is the insertion of a thiophene in the conjugation chain that not only broadens the spectral width, affecting light harvesting properties, but also increases the donor-acceptor distance, spatially affecting the HOMO-LUMO couplings and therefore electron injection and coupling to the titania.^217^ The thiophene can be inserted within the donor, extending its π system. In this case, its influence on the spectral width is much larger (30 vs. 5 nm). Modification of the anchoring group that has the dual role of a physical anchor and an electron acceptor part of the dye molecule is performed with transparent dyes that mainly absorb in the UV part of the spectrum. Large effects on electron injection and back reaction were observed. Molecular modelling reveals that with the increase of the electron affinity of the anchor, the dipole moment varies and it increases the electronic decoupling of the HOMO and the LUMO, which in turn helps electron injection in TiO_2._^218^ In contrast, the regeneration process is found independent of the dye structure modification at the interface. A series of fluorenes dyes with different electron donor and acceptor units was investigated too. The Moser group found that diphenylamine donor groups attached in the meta position increase the energy of the HOMO, stabilizing the cations of the dyes. π-extension of the core of the dye contributes to the cation stabilization as well.^20^ To summarize, the Moser group observed a direct relation of the transfer dynamics and photovoltaic performances of the dyes following their structural modifications and could highlight the importance of molecular design in DSSC development. They used it not only to tune their ability to harvest the sunlight by modifying their spectra, but also took in consideration of modifications induced in their electronic landscape, affecting the charge-transfer processes.

### D. Photoinduced charge transfer in photocatalytic systems

Intermolecular charge transfer is a crucial step for many photocatalytic reactions. It is an essential process in multicomponent photocatalytic systems which combine the photosensitizer and the catalyst as unbound molecules in the same solution. The use of a multicomponent strategy gives a wide range of freedom for the independent optimization of components of the photocatalytic system. Therefore, most of the research on the molecular photocatalytic system, for example, for water splitting and hydrogen evolution, are focused on the multicomponent approach.^219^ The time-range, in which the charge transfer occurs and the corresponding intermediates live, spans from ∼100 ns to microseconds. It is slow because the diffusion of components limits the speed of the process and it depends on their concentrations. To understand and then to optimize the catalysis, it is important to know (i) the location of the transferred charge, for example, whether it reaches the metal center of the catalyst or (ii) how the structure of the catalyst changes after the electron transfer. Below, we demonstrate how time-resolved X-ray absorption spectroscopy addresses these questions for a multicomponent photocatalytic system for H_2_ evolution. Photosensitizers used in such systems have been studied extensively using time-resolved XAS during the last few years,^220–222^ and such research was summarized in a recent review.^223^ Time-resolved XAS combines optical laser excitation and probing of the sample solution with X-ray pulses from the synchrotron. Details about a few recent experimental developments for experiments in the nanosecond-to-microsecond time ranges are described in Refs. 21 and 224. The main advantages of X-ray absorption spectroscopy are element-selectivity and high sensitivity to the electronic structure of the studied chemical element^225^ and a three-dimensional structure of its atomic environment.^220,226^ In the recent studies, the van Bokhoven group has considered the multicomponent systems which consist of the photosensitizer [Ru(bpy)_3_]^2+^, the electron relay methyl viologen and the catalyst cobaloxime Co(dmgBF_2_)_2_.^21,22^ In the initial state, the oxidation state of the Co center of the catalyst is +II. The main process, which is important for catalytic H_2_ evolution, is the electron transfer from the photosensitizer to the catalyst with the formation of Co^+^. It can occur either directly from the excited photosensitizer

$${\text{PS}}^{*}{+\text{Co}}^{2+}\to {\text{PS}}^{+}{+\text{Co}}^{+}$$ (7)

or in two steps: first the excited state of the photosensitizer is quenched by the electron relay and then the electron relay delivers the electron to the catalyst

$${\text{PS}}^{*}+\text{ER}\to {\text{PS}}^{+}{+\;\text{ER}}^{-},$$ (8)

$${\text{ER}}^{-}{+\;\text{Co}}^{2+}\to {\text{ER}+\text{Co}}^{+}.$$ (9)

Nevertheless, after the reaction step (8), the catalyst can directly interact with an already oxidized photosensitizer. In this case, the charge transfer occurs in the opposite direction and Co^3+^ is formed:

$${\text{PS}}^{+}{+\;\text{Co}}^{2+}\to {\text{PS}+\text{Co}}^{3+}.$$ (10)

In the literature, it was suggested on the basis of transient optical spectroscopy data that such charge transfer processes have similar probability, but have slightly different kinetics in the microsecond range.^227^ The sensitivity of Co K-edge X-ray absorption near-edge structure (XANES) spectra to the oxidation state of Co has been used to directly monitor such changes. Measurements of reference compounds and theoretical simulations suggest that the absorption edge shifts to higher energies in the case of Co oxidation and to lower energies in the case of reduction. In the transient X-ray absorption spectrum for different delay times from 500 ns to 50 *μ*s, the van Bokhoven group has observed a positive peak at 7.72 keV, which indicates that the absorption edge shifts to lower energies after photoexcitation. This indicates that only Co(I) intermediate is formed. The principal component analysis of the whole series of time-resolved spectra using the approach developed previously^228^ has confirmed the presence of only one intermediate on the quantitative level.

**FIG. 17.**
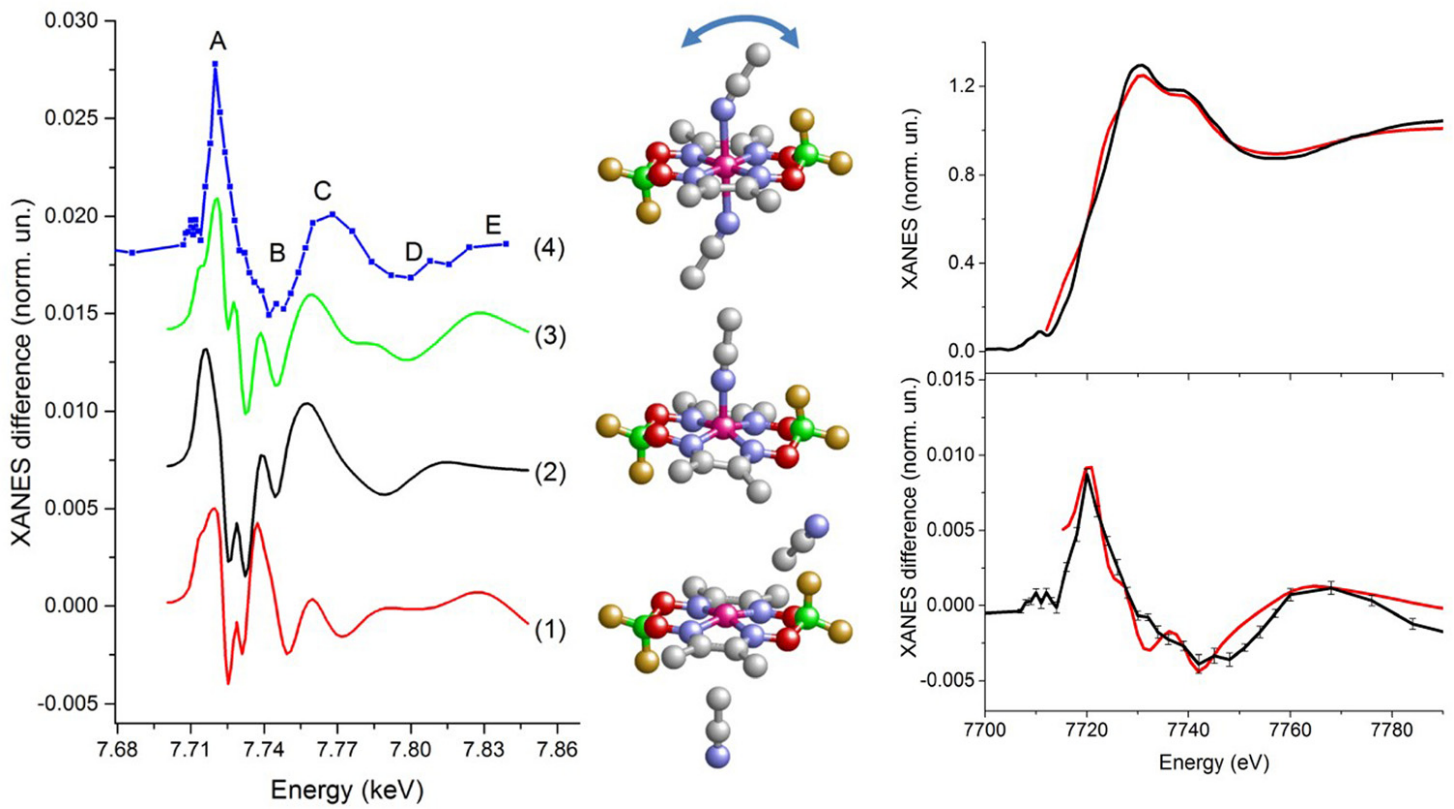
Left panel: Transient X-ray absorption spectra corresponding to the transition from the Co(II) to Co(I) state. Calculations for different models of Co(I) state: 5-coordinated model [red curve (1)], 4-coordinated model [black curve (2)] and 6-coordinated model with moderate out-of-plane displacement of Co and disordered solvent molecule [green curve (3)] are compared with the experimental data for the multicomponent photocatalytic system with the Co(dmgBF_2_)_2_ catalyst (blue curve 4). Central panel: Structural models used for the calculations. Grey atoms are C, Blue: N, Green: B, Yellow: F, Red: O, and Magenta: Co. H atoms are omitted for clarity. Right panel: Comparison of the experimental Co K-edge XANES spectra of cobaloxime in acetonitrile (black line on the top) with the theoretical simulation for the best-fit structure (red line on the top). Calculated transient XANES data for the best-fit model (red line, bottom) are compared with the experimental data (black line, bottom). Adapted with permission from Smolentsev *et al.*, Faraday Discuss. **171**, 259 (2014). Copyright 2014 The Royal Society of Chemistry, licensed under CC BY 3.021 and adapted with permission from G. Smolentsev and V. Sundstrom, Coord. Chem. Rev. **304**–**305**, 117 (2015). Copyright 2015 Elsevier.^223^

Then, the van Bokhoven group has used the theoretical analysis of transient XANES data to determine the structure of the Co(I) intermediate. As a first step, they have compared a few models at the qualitative level. Using a DFT four-coordinated model of Co(I) has been suggested in the literature.^229^ The five-coordinated structure has been constructed based on XRD data.^230^ In both these models, Co is displaced significantly (0.25–0.27 Å) out of the plane formed by dmg^−^ ligands. Six-coordinated model with the distorted arrangement of Co has been suggested starting from the Co(II) structure and moving atoms in the direction towards the 5-coordinated structure, but with smaller displacements (0.14 Å).^21^ Theoretical XANES spectra of these models have been calculated using full multiple scattering theory.^231^ Simulated transient XANES spectra are compared with the experimental ones in Fig. 17, left. Best qualitative agreement with experiment has been obtained for the 6-coordinated model. As a next step, the van Bokhoven group has refined this model using quantitative XANES fitting. They have varied the distances between Co and the nearest solvent molecules, the distance between Co and the dmg^−^ groups and moved Co out of the plane formed by N atoms of the dmg^–^ groups. In the best-fit model, one bond with the solvent remains the same as in the Co^2+^ state, while another became weaker with 0.11 Å increase of the bond length. Co atoms move out of the plane by 0.08 Å. The theoretical spectrum of this model agrees with the experiment (right panel of Fig. 17).

## VI. CHARGE TRANSFER TO SOLVENT

Aqueous halide anions exhibit intense discrete absorption bands in the deep ultraviolet (UV) below 270 nm.^232^ Aqueous isoelectronic divalent atomic anions (S^2−^, Se^2−^, and Te^2−^), and in some solvents, alkalides such as Na^−^, K^−^, and Rb^−^ exhibit similar bands at longer wavelengths.^233–235^ These bands are due to the so-called, charge-transfer-to-solvent (CTTS) states, which arise from the interaction between the ions and the solvent, usually water. They represent excited states of the solute-solvent system, which do not exist in the gas phase. The solvent around the ion is fluctuating and strongly inhomogeneous, so that the energy of CTTS states must reflect the distribution of environments around the solute in the ground state. Excitation of CTTS states leads to electron release into the solvent, but because these are excited states of the solute-solvent system, when the former is an atomic ion (i.e., lacking internal degrees of freedom), the dynamics of electron transfer to the solvent is entirely determined by the solvent species. In this respect, CTTS states are ideal objects to investigate the process of purely electronic solvation dynamics. CTTS states are not limited to atomic ions, but also occur in molecules, and the case of aqueous ferrous cyanide [Fe(CN)_6_]^4+^ is the most famous example, with its CTTS states lying in the 260–295 nm range and therefore, easily accessible.^236^ Here, we focus on the studies of aqueous iodide and ferrous hexacyanide by the Chergui group.

**FIG. 18.**
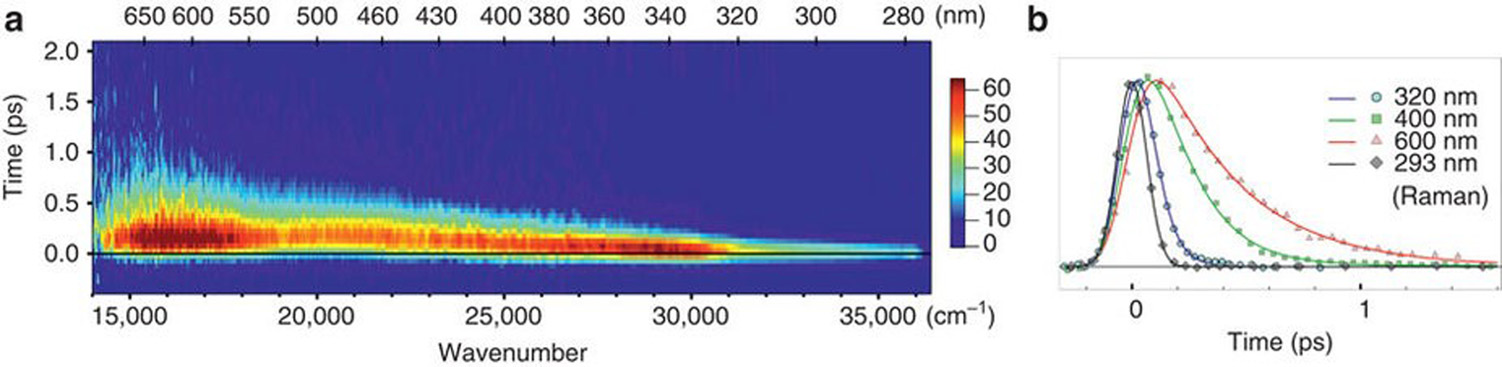
Femtosecond fluorescence of aqueous iodide. (a) Fluorescence of 1 M NaI dissolved in water upon 266 nm excitation. The Raman signal from water was removed from the plot. (b) Normalized kinetic traces at different wavelengths with their representative fits (continuous lines), compared with the Raman signal from water at 293 nm, whose temporal width gives the instantaneous response function of the setup. Reprinted with permission from Messina *et al.*, Nat. Commun. **4**, 2119 (2013). Copyright 2013 Macmillan Publishers Ltd.^24^

Of all halides, aqueous iodide has long been the prototype system for this type of study because its lowest CTTS band rises at approximately 260 nm and peaks near 240 nm. The key questions the Chergui group has been addressing in recent years are as follows: (a) How is the electron release into the solvent affected by the structure of the solvent shell prior to excitation? (b) How is the electron release affected by the solvent rearrangements induced by excitation, and on what time scales? (c) What is the role of intramolecular degrees of freedom in the CTTS dynamics? One of the main issues in addressing these questions is the type of observables one uses. Most studies of CTTS dynamics have focused on the detection of very strong absorption of the solvated electron, which is very broad with a maximum near 800 nm, and more recently by ultrafast photoelectron spectroscopy of liquid jets.^232,237–244^ These methods are exclusively sensitive to the electron signal, that is, to one of the final products of the CTTS dynamics as the observation of the halogen atom has always been elusive. The fact that the absorption by the CTTS states is close to that of the solvated electron makes it impossible to specifically address their dynamics.^232^ The above studies showed that in water, the electron is detached from iodide within 0.2 ps and relaxation of the host solvent cavity ensues (in about 1 ps) to form a solvated electron. In Refs. 23, 245, and 246, the Chergui group addressed the fate of the solvation shell around the newly formed iodine species using ps and fs X-ray absorption spectroscopy and found that the solvation shell turns from a hydrophilic to a hydrophobic interaction with the solute within 3 ps of electron ejection. In order to address the step of electron ejection itself during the CTTS dynamics, the Chergui group used ultrafast fluorescence detection since it provides a direct measurement of the electron departure to the solvent, because photons are emitted as long as there is still an overlap of the ground and excited (CTTS) state wavefunctions. The challenge is to be able to detect ultrashort fluorescence signals in the deep-UV,^247^ and this was reported for the first time in the case of aqueous I^−^.^24^ The fluorescence spectrum of aqueous iodide, measured upon excitation at 266 nm, is shown in Fig. 18, as a function of time. An emission spanning from the deep-UV (approximately 300 nm) to the visible region (approximately 670 nm) appears promptly at time zero. Its decay is strongly wavelength-dependent going from about 60 fs at *λ* < 330 nm to ∼400 fs at 650 nm. These results reveal the very large inhomogeneity of the excited centres with each excited iodide centre having a somewhat different solvent shell upon excitation. The starting configuration determines the subsequent dynamics where the solvent accommodates the excited species giving rise to very different Stokes shifts spanning almost 1 eV. The redder the emission, the larger the Stokes shift, therefore, the more stable the departure configuration, which is reflected in a longer decay time. Various time scales, in particular, in the redder part of the spectrum could not be observed by TA or other methods because the signal of the solvated electron overshadows these dynamics.

In the case of aqueous [Fe(CN)_6_]^4−^, excitation below 310 nm leads to the oxidation of the system, which were unambiguously identified by ps XAS.^248^ However, surprisingly, attempts to observe the CTTS fluorescence in this case failed, which either points to an extremely fast process and/or that the electron ejection to the solvent adopts a route that is optically silent.

## VII. INTER-SITE CHARGE TRANSFER IN SENSITIZED TRANSITION METAL OXIDES

Of crucial importance for solar energy conversion,^249^ photocatalysis^250,251^ and surface chemistry^252,253^ are processes where charges are either transferred to the substrate from an adsorbate or the reverse. The identification of the terminal site of the electron transfer is also crucial as their structure and energetics determine the outcome of the subsequent processes or reactions.^250,254,255^ In relation to the use of bare or sensitized transition-metal oxides (TMOs) in solar energy conversion and photocatalysis, it is important to determine the fate of charge carriers with elemental selectivity and time resolution, following the delivery of charges to the conduction or valence band, either by excitation above the band gap of the material or by excitation of the sensitizer.

**FIG. 19.**
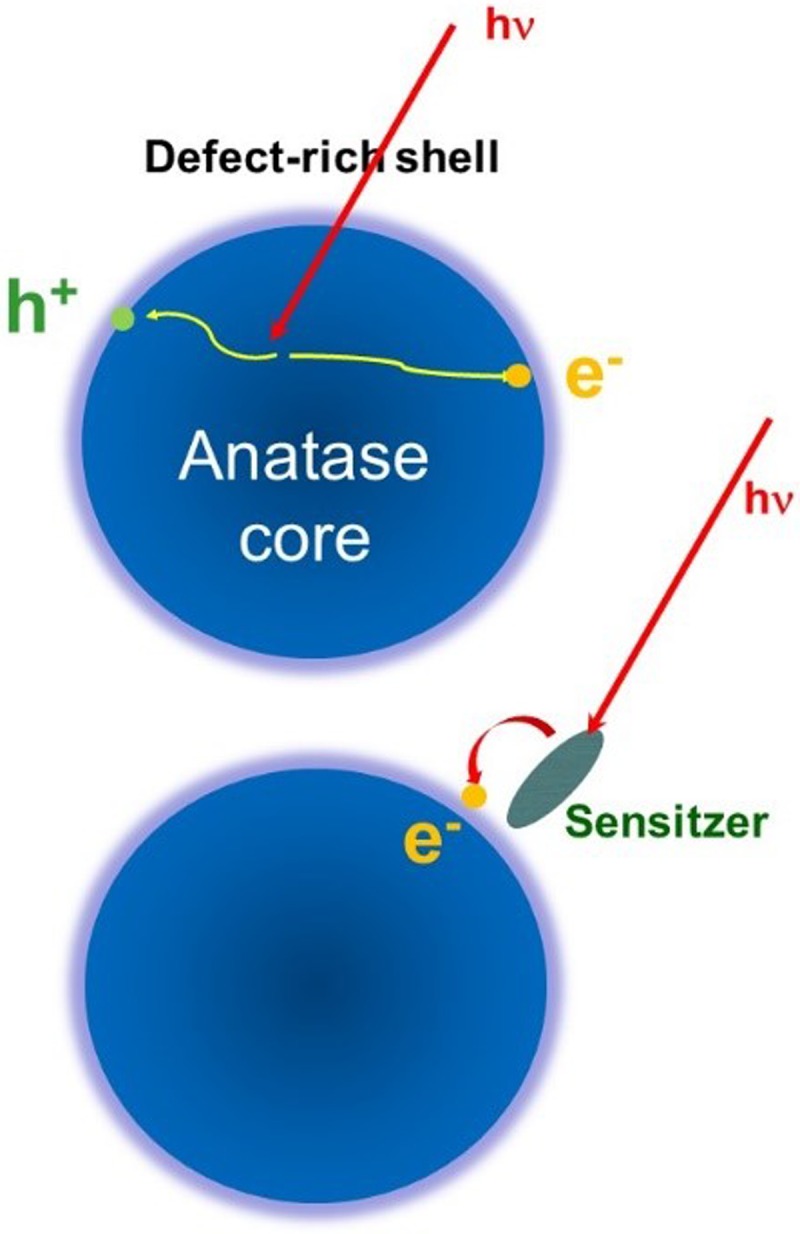
(Top) Above band-gap excitation of TiO_2_ nanoparticles leading to the trapping of charge carriers in the sub-surface region of the defect-rich shell. (Bottom) Excitation of the dye sensitizer leading to the trapping of the electron at the outer surface of the defect-rich shell.^25,26^

**FIG. 20.**
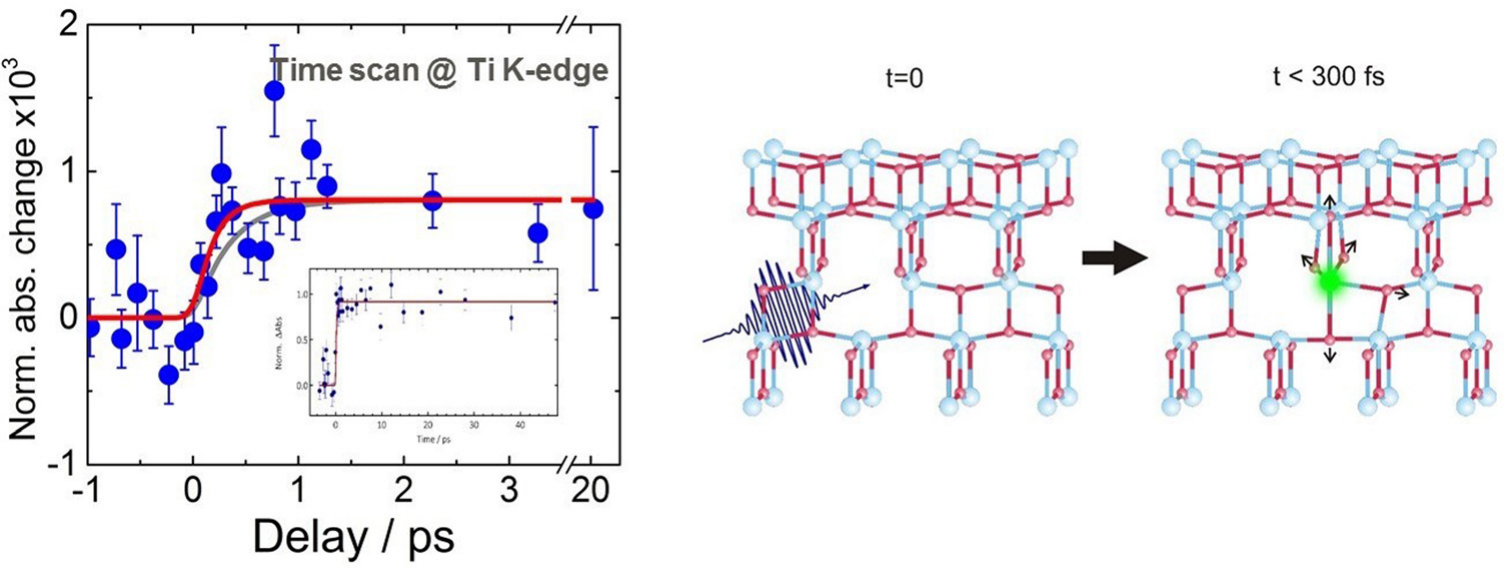
(Left) Femtosecond optical pump/Ti K-edge X-ray absorption probe of bare TiO_2_ nanoparticles excited above the band gap. Reprinted with permission from Santomauro *et al.*, Sci. Rep.-Uk. **5**, 14834 (2015). Copyright 2015 Macmillan Publishers Ltd., licensed under CC BY 4.0. The time trace shows an ultrafast localization of the electron at a Ti atom in about 170 fs (red trace), but the large error bars imply a rise time of as long as 300 fs (grey trace). (Right) Schematic description of the electron trapping at pentacoordinated defects. The implication of the fs XAS result is that the electron is trapped at or in the immediate vicinity of the unit cell where it is created.^26^

Time-resolved X-ray absorption spectroscopy is the tool of choice in this case.^256^ It was implemented at the Fe K-edge with 100 ps resolution by Gilbert and co-workers^257,258^ who probed the fate of the electron in iron (oxyhydr)oxide after injection from an adsorbed dye and concluded about the formation of small electron polarons. The Chergui group carried out a comparative study of bare and dye-sensitized anatase and amorphous TiO_2_ nanoparticles at the Ti K-edge with 70 ps resolution^25,26^ and concluded that (a) in all cases, the electron gets fully trapped at the Ti centre of a defect and leads to the formation of a small polaron and (b) the nature of the trapping site is different upon band gap excitation and injection. In the former case, it was concluded that the electron is trapped deep in the defect-rich surface shell, while in the injection case, the electron is trapped at the outer surface (Fig. 19). The latter is in line with the hypothesis of a charge-transfer exciton formed between the trapped electron and the cationic dye at the surface, which was inferred from THz studies by the Moser group;^259^ and (c) the recovery kinetics of all traps exhibits a clear biexponential decay with time constants being longer for the injection case compared to the band gap excitation. This is due to the fact that the latter yields holes in the valence band that favour a faster electron-hole recombination. Remarkably, the biexponential kinetics was also found in the case of bare and dye-sensitized amorphous nanoparticles (NPs) with time constants that were quite close to those of the bare anatase NPs. The fact that only two components show up in these measurements along with the spectroscopic signatures that appear in the pre-edge region led the Chergui group to conclude that the most likely trapping sites are pentacoordinated ones due to inequivalent oxygen vacancies. The next issue was to determine the trapping time of the electron at a defect, and for that matter, the Chergui group carried out a femtosecond Ti K-edge absorption experiment using the slicing scheme at the Swiss Light Source (SLS).^26^ The results are shown in Fig. 20 for the above band gap excitation. It was found that the trapping of the electron occurs in less than 200 fs, implying that it is trapped at or near the unit cell where it is created. This trapping time was later confirmed by ultrafast electron diffraction studies of the Zewail group^260^ on photoexcited titanosilicate materials consisting exclusively of pentacoordinated Ti centres. Very recently, Obara *et al.*^261^ repeated the experiment described in Ref. 26, but this time with X-ray pulses from the free electron laser. The increased signal-to-noise ratio allowed them to record the kinetics at the pre-edge, which reflects the symmetry of the traps, at the edge, which reflects the oxidation state, and above the edge, which reflects the geometry. Interestingly, they found that the pre-edge and above edge signals showed the same rise of ∼330 fs, while the edge shows a fast rise of about 100 fs. These results show that electron trapping and reduction of a Ti^4+^ centre takes place much faster than the ensuing structural relaxation around the reduced atom. The ultrafast trapping inferred from these studies^26,261^ implies that the electrons generated by the above band-gap excitation do not have time to migrate and therefore, in the present studies, they are mostly those created in the surface shell.

The above approach based on time-resolved XAS, while providing an unambiguous marker of the electron in the TMO, is mostly sensitive to trapped charges. However, most charges are not trapped, and the universality of this approach is questionable when no electron trapping occurs, which is most likely the case in ZnO, another very popular solar material. Indeed, a recent work on the latter system by Milne *et al.*^262^ shows that the response of hole trapping in the valence band can be monitored by looking at the signal of the metal K-edge, due to a strong distortion that occurs around hole traps.

Additionally, it would be useful to have a lab-based approach for monitoring charge injection in real time. The past twenty years have seen the implementation of several methods based on the use of THz to visible light, which can visualize the free charge carriers in the conduction band. This free-carrier absorption is described by the Drude model and is unspecific to the substrate. Thus, the use of such probes is problematic when dealing with solid-state sensitizers, as the free carrier absorption of the latter and that of the substrate can no longer be distinguished. In a recent study, the Chergui group investigated the nature of excitonic transitions in anatase TiO_2_, which is an indirect bandgap material, and found that the first exciton, centred at 320 nm, is a strongly bound exciton intermediate between a Frenkel and a Wannier exciton.^263^ Given the broad-band 2D UV capabilities of the Chergui group,^264,265^ there is now the possibility to pump with variable energies (between 250 and 380 nm or in the visible region between 500 and 760 nm) below, at and above the optical band gap and to probe with a white-light continuum in the 250–380 nm range. This unique capability has been implemented for the first time to investigate the fate of the excitonic transitions in TiO_2_ and ZnO.^266^ Using ruthenium N719 dye-sensitized metal oxide nanoparticles, the Chergui group found clear signatures of injection in the excitonic response in these two materials: in TiO_2_, the response is dominated by Coulomb screening with a weak contribution of Drude-like absorption, while in the case of ZnO, the response is mainly due to phase-space filling. Importantly, in both cases, the kinetics of injection perfectly reproduce those measured by THz to visible radiation. This proof-of-principle demonstration of a deep-UV substrate specific probe of a charge injection now opens the door to implementation of this method in systems consisting of solid sensitizers, such as perovskites or gold nanoparticles.

## VIII. CONCLUSIONS AND OUTLOOK

This review summarizes recent results on the transfer of charge across molecular and condensed-matter systems on time scales ranging from attoseconds to microseconds. These results have been obtained with state-of-the-art or even newly developed experimental techniques and methodologies at the forefront of time-resolved spectroscopy. This review deliberately focuses on the progress achieved in understanding the elementary steps of charge transfer, rather than on the development of experimental methods, although the latter was a crucial aspect in most of the experiments. These results show the considerable potential of time-resolved spectroscopy with ever higher temporal resolution to isolate the true quantum-mechanical elementary processes underlying complex transformations. This potential is still far from being fully realized and is likely to yield fundamentally new insights in the years to come.

In particular, the importance of the purely electronic phenomenon of charge migration within the overall process of charge transfer needs to be established. The key questions in this respect concern the role of electronic coherence in charge transfer, the lifetime of such electronic coherences in the presence of nuclear motion and their effect on the unfolding nuclear dynamics. Once these phenomena are understood, the control over electronic coherences on the attosecond time scale might offer a new approach to controlling charge-transfer processes taking place on much longer time scales. Addressing these important questions will however require further developments in experimental techniques, such as the extension of attosecond laboratory sources to the X-ray domain to make them applicable to complex systems and the improvement of time resolution at X-ray free-electron laser facilities.

Rapid progress in the development of high-harmonic sources^267–270^ has indeed recently enabled the first time-resolved measurements in the soft-X-ray domain.^271,272^ Similarly, rapid advances in Free-Electron-Laser sources^273,274^ are likely to bring few- to sub-femtosecond resolution into reach,^275^ at least in the hard X-ray domain. These new developments add the element-specificity of X-ray spectroscopy to the tools of attosecond/femtosecond spectroscopy, which will enable element-specific studies of charge migration and charge transfer.
